# Effects of Serotonin Transporter Genotype and (Un)Predictable Maternal Separation on Affective, Cognitive, and Social Behaviors in Female Rats

**DOI:** 10.1111/gbb.70069

**Published:** 2026-10-12

**Authors:** Danielle J. Houwing, Mayerli A. Prado‐Rivera, Jenny Borkent, Marrit Gerbrandy, Chantal Arling, Jocelien D. A. Olivier

**Affiliations:** ^1^ Neurobiology, Groningen Institute for Evolutionary Life Sciences University of Groningen Groningen the Netherlands

**Keywords:** behavior, development, early life stress, gene × environment, offspring, serotonin transporter

## Abstract

Serotonin transporter (SERT) variation is linked to depression risk after stress, conferring an increased risk to SERT‐linked polymorphic region (5‐HTTLPR) short‐allele carriers, who show heightened sensitivity to cumulative stress and childhood maltreatment. To elucidate the neurobiological mechanisms underlying SERT × early‐life adversity interactions, animal models capturing this genetic vulnerability are of translational relevance. We evaluated four maternal separation (MS) paradigms to determine how different forms of early‐life stress influence adult behavior in female SERT‐deficient rats, given the higher prevalence of depression in women and the limited translational value of male‐only preclinical findings. We applied several behavioral tests to wildtype (SERT^+/+^), heterozygous (SERT^+/−^), and homozygous (SERT^−/−^) knockout females exposed to MS: predictable 3 h/day (MS180) or 6 h/day (MS360), unpredictable 3 h/day (MSU180), or unpredictable 3 h/day plus unpredictable maternal stress (MSUS180). Controls were 15‐min handled. SERT^−/−^ females exhibited higher anxiety‐like responses, passive stress coping, and increased social exploration. MS180 and MS360 induced some anxiety‐like behaviors, while MS360 increased hedonic responding. Females responded differently to MS depending on their SERT genotype. First, some anxious behaviors of SERT^−/−^ females were attenuated when exposed to unpredictable MS (MSU180 and MSUS180). Second, MSU180 reduced object recognition in SERT^−/−^ females, while enhancing it in SERT^+/−^ females. Third, SERT^+/−^ females exhibited less passive stress coping when exposed to MS360 than all other groups. Neither predictable nor unpredictable MS exposure increased vulnerability to depressive‐related behaviors in SERT‐deficient females. Variations in SERT expression may confer some beneficial effects in response to MS, depending on the specific genotype and behavioral domain evaluated.

## Graphical abstract

Female rats of three serotonin transporter (SERT) genotypes were exposed to maternal separation paradigms modeling early life stress, followed by adult behavioral testing. Maternal separation did not consistently induce depression‐related behavioral phenotypes in SERT‐deficient females. Instead, SERT genotype modulated the behavioral consequences of early‐life stress in a genotype‐ and domain‐specific manner.

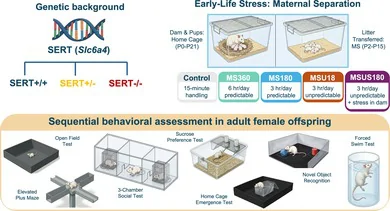

## 1 Introduction

Depressive disorders are among the most debilitating mental conditions worldwide, with major depression disorder accounting for the highest prevalence [1], and exhibiting high comorbidity with anxiety disorders [2]. Global estimates continue to rank depression as a leading cause of years lived with disability [3]. Cumulative evidence associates a higher risk of major depression with both environmental and genetic factors [4]. In fact, there is an increasing consensus that gene–environment (GXE) interactions, rather than single risk factors, critically shape individual vulnerability and resilience to depression [5, 6, 7]. The most studied GXE interaction was first described by Caspi et al. [8], who showed that the risk of developing depressive symptoms increased along with the accumulation of stressful events, depending on variation in the serotonin transporter (SERT) gene. Within the serotonin‐transporter‐linked promoter region (5‐HTTLPR), there are different lengths of GC‐rich repeat elements [9]. A deletion or insertion in the 5‐HTTLPR gives rise to the 14‐repeat short (S, low expressing) and 16‐repeat long (L, high expressing) alleles [9]. Caspi et al. found that individuals exposed to stress who carried one or two copies of the S allele were more vulnerable to developing depressive symptoms than L‐allele carriers [8]. Subsequent primary studies synthesized in several meta‐analyses have provided partial support for this GXE interaction [10, 11, 12]. However, other meta‐analyses have failed to replicate the effect entirely [13, 14] or have concluded that the effect sizes are modest and context‐dependent [15]. This lack of consensus is also reflected in a large‐scale individual cohort study, which found no evidence of this GXE interaction effect, suggesting that previous positive associations may be false positives [16]. In particular, early‐life adversity, including childhood trauma, abuse, neglect or chronic family stress, has been consistently associated with an increased risk for depression [6, 17, 18], although the specific contribution of the 5‐HTTLPR to these outcomes remains debated [19].

Rodent models offer a unique opportunity to uncover the neurobiological mechanisms underlying the 5‐HTTLPR × stress interactions associated with the onset of depressive symptoms. Although rodents do not carry the 5‐HTTLPR polymorphism, both mice [20] and rats [21] have been genetically modified, such that SERT heterozygous (SERT^+/−^) knockouts exhibit reduced SERT expression, while SERT homozygous (SERT^−/−^) knockouts lack SERT expression entirely. While SERT^−/−^ animals show a robust neurobehavioral phenotype (for a review see: [22]) and are still of interest for understanding the effects of extreme SERT disruption, SERT^+/−^ rodents are more neurochemically comparable to human S‐allele carriers, exhibiting reduced SERT expression and function [20, 23]. Accordingly, SERT^+/−^ models are increasingly regarded as translationally relevant tools to study genetic vulnerability to stress‐related affective disorders [24].

To model early‐life adversity such as neglect or inconsistent caregiving, early‐life stress (ELS) induced by maternal separation (MS) has been extensively used in rodents. Typically, MS involves the daily separation of pups from the dam during the first two or three postnatal weeks. Research in wildtypes has shown that MS is associated with anhedonia, the reduced ability to experience pleasure (commonly tested in the sucrose preference test), and passive stress coping (exhibited as increased immobility in tasks like the forced swim test), both of which are behaviors indicative of a depressive‐like phenotype. These outcomes have been observed across various protocols including predictable 180‐ to 240‐min daily separations relative to undisturbed controls [25, 26], 360‐min daily separations relative to briefly handled controls [27], and unpredictable 180‐min daily separations combined with maternal stress compared to undisturbed controls [28]. One study in SERT^+/−^ female rats exposed to a predictable 360‐min daily MS paradigm showed reduced sucrose preference compared with non‐stressed, 15‐min handled SERT^+/−^ females [29]. However, these animals did not exhibit increased anxiety‐like behaviors or passive stress‐coping. Importantly, the lack of comparison with SERT^+/+^ or SERT^−/−^ females precluded conclusions about genotype‐specific vulnerability to depression. In addition, it was not possible to determine whether MS exposure alone was sufficient to induce depressive‐like behaviors, regardless of the SERT genotype [29]. Another study comparing male SERT^+/+^, SERT^+/−^ and SERT^−/−^ rats found reduced passive stress coping in SERT^+/−^ but not in SERT^−/−^ males following a predictable 180‐min daily MS paradigm relative to briefly handled controls [30]. Together, these findings suggest that the behavioral consequences of MS depend on both sex and SERT gene dosage.

Despite its widespread use, MS does not consistently induce depressive‐like and anxiety‐like behaviors in rodents [31, 32]. Variability in outcomes may be partly explained by differences in maternal behavior elicited by the separation procedure. A first critical factor is the amount of time that the pups are separated from the dam. Short‐time MS (< 1 h/day) produces variable effects on maternal care, whereas longer separations (1–6 h/day) are more consistently associated with increases in specific maternal care behaviors such as licking/grooming or the time spent on the nest [33]. A second factor concerns the dams' ability to anticipate predictable separations and adjust caregiving accordingly by increasing care before and after MS [28]. To prevent such anticipation, unpredictable MS, achieved by varying the daily onset time of the separation, combined with stress exposure in the dam (maternal stress), has been shown to produce more persistent behavioral alterations in the offspring [28]. These findings highlight maternal care as a key mediator of long‐term MS effects on offspring physiology and behavior [34].

In humans, depressive disorders are nearly twice as prevalent in women as in men [35, 36], a sex difference observed across ages and world regions [37, 38]. Maternal depression during pregnancy and postpartum has been associated with adverse developmental outcomes in offspring [39, 40]. Nevertheless, the majority of rodent studies on depression have been conducted in males [32], which cannot account for the sex‐specific influence of brain anatomy, neurochemistry and hormonal functioning in mediating how depressive symptoms develop and manifest [41]. This male bias limits translational validity, particularly for disorders that disproportionately affect women. Female rodent models help determine whether depressive‐like phenotypes are sexually dimorphic, and enable the investigation of variability in disorder presentation and comorbidity patterns, as observed in humans [42]. Furthermore, female rodents provide a unique opportunity to explore the effects of maternal depression during pregnancy and the postpartum period on offspring development and behavior. Overall, the inclusion of female subjects is essential for advancing research on the neurobiology of depression.

The present study aimed to examine whether SERT genotype interacts with ELS induced by MS in the emergence of depressive‐like and anxiety‐like behaviors in adult female rats. To do so, we assessed affective, cognitive, and social behaviors. Specifically, we investigated whether (i) SERT dosage (SERT^+/+^, SERT^+/−^, SERT^−/−^) moderates the behavioral effects of maternal separation, and (ii) different types of MS (duration, predictability, and maternal stress) differentially influence these outcomes. We hypothesized that MS would induce more depressive‐like and anxiety‐like behaviors in adulthood, and that these effects would be most pronounced in genetically vulnerable animals (SERT^+/−^ and SERT^−/−^). Furthermore, we hypothesized that unpredictable MS combined with maternal stress (MSUS180) would produce the strongest depressive‐like and anxiety‐like phenotypes, reflecting a heightened disruption of maternal care. Finally, we expected genotype‐dependent differences in sensitivity to MS, consistent with the sex‐specific G × E vulnerability observed in humans.

## 2 Materials and Methods

### 2.1 Animals

The SERT knockout rat (Slc6a41Hubr) was generated by target‐selected ENU‐induced mutagenesis on a Wistar (Wistar/Crl) background (for detailed description, see [21]). All breeding took place in our own facility. In total, 49 adult SERT^+/−^ female and 35 adult SERT^+/−^ male rats were used to breed SERT^+/+^, SERT^+/−^ and SERT^−/−^ offspring. Animals were socially housed in Makrolon Type 4 cages (55.6 × 33.4 × 19.5 cm), unless stated otherwise, under standard laboratory conditions with a reversed 12 h:12 h light/dark cycle (lights off at 8:00), 40%–60% room humidity, and ad libitum access to food (RMH‐B, AB Diets; Woerden, The Netherlands) and tap water, unless stated otherwise. For enrichment, wooden gnawing sticks (10 × 2 × 2 cm) were provided inside home cages. Behavioral testing took place during the dark phase, between 9:00 and 18:00. All experimental procedures were approved by the Groningen University Committee of Animal experiments (DEC6936A) and were conducted in full compliance with the EU Directive (2010/63/EU).

### 2.2 Maternal Separation and Maternal Stress

Dams were checked daily for pup delivery, which was set as postnatal day 0 (P0). On P2–P15, pups were maternally separated as a whole litter daily during the dark phase of the reverse light/dark cycle. Litters were assigned to one of four conditions: predictable 3 h/day (MS180), predictable 6 h/day (MS360), unpredictable 3 h/day (MSU180), or unpredictable 3 h/day combined with unpredictable stressors applied to the dam while the pups were away (MSUS180). For the unpredictable groups (MSU180 and MSUS180), unpredictability was achieved by varying the daily onset time of the separation to prevent anticipation. All dams were maintained in the home cage during the separation of the litter, except for the MSUS180 group in which dams were exposed to either 20‐min restraint stress or 5‐min forced swimming in cold water. For restraint stress, dams were put inside a plastic tube in which they could move their paws but not turn around. For the forced swimming, dams were placed into a cylindrical Plexiglas tank filled up to 30 cm with water at 18°C. For control groups, pups were separated from their mother for 15 min and handled briefly to control for the confounding effects of procedural handling and nest disturbance inherent to the experimental protocol. During MS, whole litters were placed in preheated Makrolon Type 2 cages (P2–P8: 32°C ± 1°C; P9–15: 28°C ± 1°C).

At P21, pups were weaned, and ears were punched for identification and genotyping (for genotyping protocol see: [43]). Offspring of mixed genotypes were caged (3–4 per cage) with same‐sex and MS‐type mates from different litters for the rest of the study. Only females were assessed for adult behavior in this study (see Figure 1 for experimental timeline). Total number of females per group was: SERT^+/+^ (CTR = 10; MS360 = 9; MS180 = 9; MSU180 = 7; MSUS180 = 7); SERT^+/−^ (CTR = 9; MS360 = 9; MS180 = 9; MSU180 = 9; MSUS180 = 9); SERT^−/−^ (CTR = 7; MS360 = 8; MS180 = 9; MSU180 = 9; MSUS180 = 9). The number of contributing litters for each treatment and genotype, together with the distribution of animals per litter, is provided in Table S1.

**FIGURE 1:**
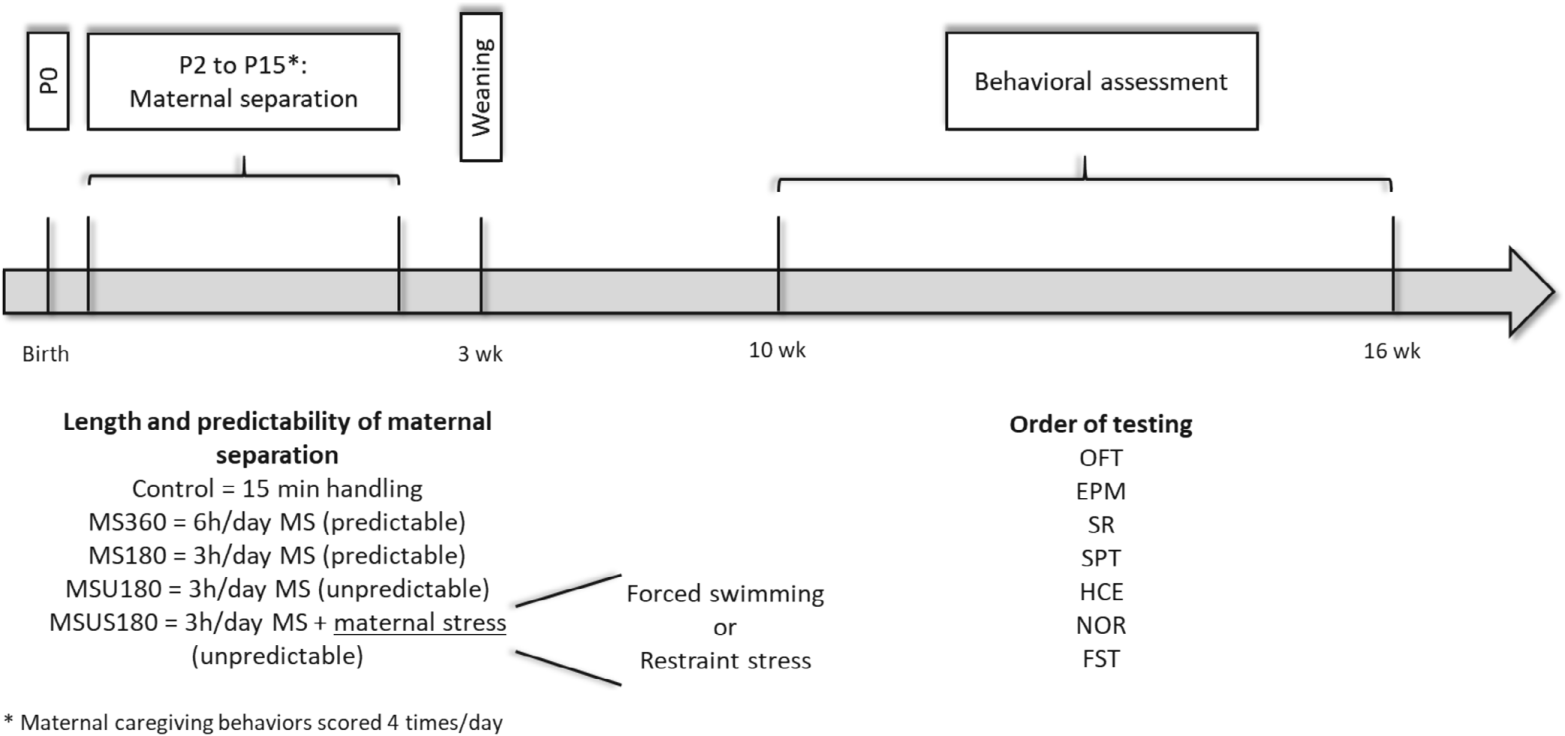
Experimental timeline of the study. SERT^+/+^, SERT^+/−^ and SERT^−/−^ female rats were exposed to different types of maternal separation (MS) or 15 min of handling (Control), and tested for behavior as adults. EPM, elevated plus maze; FST, forced swim test; HCE, home cage emergence test; NOR, novel object recognition; OFT, open field test; P, postnatal day; SPT, sucrose preference test; SR, social recognition test.

To evaluate the effect of different MS procedures on weight, we monitored dams' body weight daily from P1 to P16, and recorded offspring's body weight weekly from P7 to P70 to evaluate growth across development.

### 2.3 Maternal Care Assessment

Dams' caregiving behaviors were observed four times a day from P2 to P15. Observation sessions occurred during the dark phase of the reverse light/dark cycle, at 9:00, 11:30, 14:30, and 17:00. Each observation session consisted of 30 min, during which dam nest occupancy and activity were scored once per minute using instantaneous scan sampling (resulting in 30 1 min epochs). Dam nest occupancy was scored when the dam was within the nest she had built with the provided nesting material. Activities scored were pup‐directed behaviors (licking/grooming, nest building, and pup retrieval) and self‐directed behaviors (drinking, eating, and self‐grooming).

For each observation session, dam nest occupancy was calculated as the number of epochs the dam was observed on the nest, divided by the total number of epochs (30), multiplied by 100; then, the four observation sessions were averaged to get the percentage of each postnatal day. We averaged data from P2 to P8 (where maternal care behaviors peak and pups lack thermoregulation) to calculate the dams' nest occupancy for the first week, and from P9to P15 for the second week.

Pup‐directed behaviors within the nest were extremely challenging to observe as bedding material limited the vision; hence, when the experimenter was doubtful about what occurred within the nest, only the location of the dam was scored. Due to this limitation, dams' nest occupancy was the only maternal behavior further analyzed.

### 2.4 Adult Offspring

In total, 15 groups were behaviorally tested between the ages of 10–16 weeks. Tests were at least 1 week apart in the following order: open field test (OFT), elevated plus maze (EPM), social recognition test (SR), sucrose preference test (SPT), home cage emergence (HCE) test, novel object recognition (NOR) test, and forced swim test (FST). Estrous cycle was measured with an impedance apparatus (model MK‐11, Muromachi, Tokyo, Japan) before starting each test; females in proestrus stage were left in their home cages and checked the following days until they were not in the fertile part of the cycle, thereby minimizing behavioral variability and potential confounders driven by peak circulating ovarian hormones [44]. Therefore, only those not in proestrus were assessed.

### 2.5 Behavioral Tests

#### 2.5.1 Open Field Test

The OFT allows testing exploratory and anxiety‐like behaviors, based on the tendency of rodents to explore novel environments and avoid open spaces. Distance moved is indicative of locomotor activity, while less duration/frequency in the center area is associated with higher anxiety [45]. The open field consisted of an open square arena (100 × 100 × 40 cm), with a black wooden floor and walls. The arena was subdivided into a center (1 square of 60 × 60 cm) and outer area near the walls (20 cm wide). Females were tested under dim light conditions, and their behavior was recorded for 10 min using automated animal tracking software (EthoVision XT 11.5, Noldus Information Technology B.V., The Netherlands) to calculate the total distance moved (m) across the arena, as well as the duration and frequency in the center area.

#### 2.5.2 Elevated Plus Maze

The EPM is a widely used test to assess anxiety‐like behaviors, with fewer entries and less time spent in the open arms interpreted as indicators of increased anxiety‐like behavior [46]. Our maze consisted of 2 open (45 × 10 × 1 cm) and 2 closed (45 × 10 × 50 cm) arms opposite to each other elevated at a height of 50 cm. Females were tested under dim light conditions. Each subject was placed in the center of the maze facing an open arm and was allowed for free exploration during 5 min. Behavior was recorded using automated animal tracking software (EthoVision XT 11.5, Noldus Information Technology B.V., The Netherlands). Time spent in the open arms was calculated based on the duration the animal was detected within the predefined open‐arm zones, irrespective of whether it was actively moving or stationary. To test overall exploration, total distance moved (m) across the maze was also included.

#### 2.5.3 Social Recognition Test

The SR test assesses the ability to recognize an unfamiliar mate in comparison to a familiar one, based on the tendency of rodents to investigate novel conspecifics. Using a three‐chamber apparatus, the time spent in the chamber containing the novel conspecific is quantified as a measure of sociability (Trial 1) and social recognition (Trial 2) [47]. Our three‐chamber box (120 × 80 × 40 cm) is subdivided into 3 connected identical chambers (40 × 80 cm). First, the experimental female was placed for 10 min into the empty middle chamber for habituation, with no access to the other two chambers. Afterwards, an unfamiliar female rat (7–10 weeks old, Stranger 1) was randomly placed under a plastic grid in the left or right chamber, while an empty grid was present in the other chamber. The doors were opened, and the experimental female had access to all 3 chambers during a 10‐min testing period (the sociability trial). Afterwards, the experimental female was placed in the middle chamber again with the doors closed. A second unfamiliar female (Stranger 2) was now placed into the previously empty grid. The doors were opened again, and the experimental female was allowed to explore all chambers for 10 min (the social recognition trial). Behavior was recorded using automated animal tracking software (EthoVision XT 11.5, Noldus Information Technology B.V., The Netherlands) to calculate the total distance moved (m) during habituation, and the duration of social exploration during the sociability and social recognition trials. Social exploration was counted when the female's nose was pointing in the direction of the social stimulus at a distance of no more than 2 cm and/or by directly touching the social stimulus with the nose or forepaws. For the sociability trial, the time spent with stranger 1 and the empty grid was scored, and the sociability index was calculated as:

$$\text{Sociability index}=\left(\frac{\text{Time exploring stranger}\;1}{\text{Time exploring stranger}\;1+\text{Time exploring empty grid}}\right)$$

An index above 0.5 indicated the preference of stranger 1. For the social recognition trial, the time spent with each social stimulus was scored and the social recognition index was calculated as:

$$S.\;\text{Recognition index}=\left(\frac{\text{Time exploring stranger}\;2}{\text{Time exploring stranger}\;2+\text{Time exploring stranger}\;1}\right)$$

An index above 0.5 indicated the preference of stranger 2 over stranger 1. For a general measure of locomotor activity, total distance traveled (m) within the empty middle chamber during the habituation trial was also included.

#### 2.5.4 Sucrose Preference Test

The SPT is one of the most reliable tests to evaluate hedonic responses in rodents by leveraging their natural preference for palatable solutions. Reduced sucrose consumption is interpreted as an index of anhedonia, a core feature of depression [48]. Females were individually housed and habituated with two bottles of water, one on each side of the cage, for three consecutive days. Following habituation, animals underwent 8 days of testing consisting of alternating sucrose and water‐only days. On sucrose test days, rats were presented for 24 h with one bottle of water and one bottle containing a sucrose solution. On intervening days, both bottles contained water. Four sucrose concentrations were tested sequentially across sucrose days: 0.25%, 0.50%, 0.75%, and 1.0%. Bottle positions were alternated between test days to prevent side preference. This low, ascending concentration range was selected to evaluate shifts in baseline hedonic thresholds and prevent potential ceiling effects associated with higher sucrose concentrations. Sucrose bottle locations on the cage were alternated on sucrose days to prevent spatial bias. Fluid consumption (gram) was determined daily. The preference for sucrose over water was calculated as:

$$\text{Sucrose preference}\;\left(\%\right)=\left(\frac{\text{Sucrose solution intake}\;\left(g\right)}{\text{Total fluid intake}\;\left(g\right)}\right)\times 100\%$$

Actual sucrose intake in mg per gram of rat weight—corrected for body weight—was also calculated as:

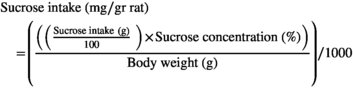




#### 2.5.5 HCE Test

The HCE test assesses neophobia, or the tendency to avoid novel objects and places. The time it takes an animal to leave its home cage serves as a measure of anxiety [49]. The HCE test was scheduled immediately after the SPT test because it requires testing females individually in their home cage. The entire home cage was moved to an adjacent test room and placed in an open field (100 × 100 × 40 cm). The lid of the home cage was removed, and a wired metal grid was placed over the edge of the cage for easier escape. Using a stopwatch, the latency to escape the home cage was measured. The female was considered to have escaped when all four paws were over the edge of the cage, either by climbing onto the grid or by jumping on top of one of the cage walls. The cutoff to escape the home cage was set at 10 min, and rats who did not escape were given that score for the latency. After this test, the subject was socially housed again.

#### 2.5.6 Novel Object Recognition

The NOR test evaluates recognition memory and is based on the tendency of rodents to explore novel objects and places. The time spent with a novel object is used as an index of its recognition. This task took place on two consecutive days and consisted of two phases: habituation and testing. For habituation, the female was placed for 10 min inside an open arena (50 × 60 × 51 cm) containing two different spatial cues posted on the walls. Twenty‐four hours later, the rat was first habituated again for 3 min, followed by the placement of two identical objects (transparent glass bottles filled with flour) in two opposite corners of the open arena for 3 min (Trial 1). After that, both the rat and the objects were removed for 45 s, after which the testing trial began (Trial 2). The 45‐s retention interval primarily assesses immediate object recognition, with relatively limited demands on longer‐term memory retention [50]. In this trial, the female was exposed to two objects placed in the same positions as Trial 1 for another 3 min; this time one object was familiar and the other was novel (an opaque purple plant sprayer). Object exploration was scored using The Observer XT 14 (Noldus Information Technology B.V., Wageningen, Netherlands) when the rat's nose was pointing in the direction of the object at a distance of no more than 2 cm or by touching the object with the nose. The exploration time of each object (obj1, obj2) during Trial 1, and the exploration time of the novel (N) and familiar (F) objects during Trial 2 were scored. The total exploration time during Trial 2 was calculated as the time exploring N plus the time exploring F, and the discrimination index (DI) of the novel object was calculated as:

$$\mathrm{DI}=\left(\frac{\text{Time exploring}\;N-\text{time exploring}\;F}{\text{Time exploring}\;N+\text{time exploring}\;F}\right)$$

A DI above zero (where 0 indicates no preference) indicated discrimination of the novel object.

#### 2.5.7 Forced Swim Test

The FST assesses the stress‐coping strategy of rodents by leveraging their natural escape response to water. The time during which an animal ceases attempts to escape and remains immobile is interpreted as a passive stress response [51]. Cylindrical Plexiglas tanks (50 cm × 18 cm diameter) were filled up to 30 cm with water at 22°C ± 1°C. During the induction phase on Day 1, the female was placed in the water for 15 min. After 24 h, behavior was scored during a 5‐min test. Mobility and immobility behavior were manually scored. Immobility was defined as making no movements for at least 2 s or making only those movements that were necessary to keep the nose above the water. Slightly moving paws or looking for support by pressing paws against the wall of the cylinder was scored as immobility, whereas active climbing, diving, and swimming along the wall were scored as mobility. Percentage immobility was calculated by dividing the total time spent immobile by the total duration of the test, and then multiplying by 100.

### 2.6 Statistical Analysis

All statistical analyses were carried out in R version 4.4.1 [52]. Results from all analyses were considered as statistically significant when *p* < 0.05.

#### 2.6.1 Analysis of Dams' Outcomes

To determine the effect of MS type on dams' body weight gain, a two‐way mixed ANOVA was performed, with day as the within‐subjects factor and MS type (CTR, MS360, MS180, MSU180, MSUS180) as the between‐subjects factor. To assess the effect of MS type on the dams' nest occupancy, two one‐way ANOVAs were conducted separately for Week 1 and Week 2. Before running each ANOVA, data were screened for extreme outliers using Tukey's IQR method with the identify_outliers() function [rstatix package v0.7.2]. A mixed‐linear model for body weight was then built using the lmer() function [lme4 package v1.1–35.3], and a linear model for dams' nest occupancy was built using the lm() function [car package v3.1–2]. Assumptions of normality and homoscedasticity of residuals were checked for each model, and ANOVAs were conducted using the Anova() function [car package v3.1–2]. Multiple pairwise comparisons were performed using Fisher's LSD test when significant main or interaction effects were found. Degrees of freedom (df) from the ANOVA are reported, and because all comparisons use the same residual df, only *t*‐statistics and *p* values are reported for the pairwise tests. When residuals deviated from normality, raw data were transformed based on the degree of skewness using square‐root (mild), log10 (moderate), or inverse (severe) transformations; the model was then updated, and the ANOVA repeated.

#### 2.6.2 Analysis of Offspring's Outcomes

To determine main and/or interaction effects of SERT genotype and MS type on offspring growth, a two‐way ANOVA per postnatal day (P7, 14, 21, 28, 35, 42, 49, 56, 63, 70) was conducted, with SERT genotype (SERT^+/+^, SERT^+/−^, and SERT^−/−^) and MS type (CTR, MS360, MS180, MSU180, MSUS180) as factors. Offspring behavioral data were analyzed using two‐way ANOVAs, with SERT genotype and MS type as factors. For all offspring outcomes, linear models were built, and the same statistical procedures as described for dams were followed.

For the NOR test, we also tested whether the DI of each group was significantly above zero (no preference) based on Akkerman's statistical recommendation [53]. We created 15 independent fictional comparison groups and conducted paired one‐sided *t*‐tests between the novel object DI of each group and the DI of its respective fictional comparison group (DI_fic_). Each fictional group had 0 as the mean; the variance was estimated by using the duration of obj1 exploration during Trial 1 because this was the first time that animals were exposed to the objects, so it was expected that the natural variability of objects' exploration was expressed without the influence of any previous experience. We created independent DI_fic_ as a variation from Akkerman's study as it cannot be ruled out that the natural variability of objects' exploration in Trial 1 was not under the influence of the SERT genotype, the MS type, or their interaction.

## 3 Results

### 3.1 Dams' Body Weight Gain

A main effect of postnatal day on dams' body weight gain from P1 to P16 was found (Figure 2; *F*_(15,315)_ = 133.11, *p* < 0.001), in which the weight gain was increasing progressively over the days, being the highest on P16. A main effect of MS type was also found (*F*_(4,21)_ = 5.16, *p* < 0.01); further analysis indicated that the weight gain was less in MSU180 (*t* = 2.33, *p* < 0.05) and MSUS180 (*t* = 3.84, *p* < 0.001) dams compared to CTR. An interaction effect of postnatal day × MS type was also found (*F*_(60,315)_ = 1.42, *p* < 0.05). From P5 to P15, MSU180 dams showed significantly reduced weight gain relative to CTR dams on multiple days, though not all reached significance, while MSUS180 dams showed significantly less weight gain than CTR dams from the first day of MS (P2) until the last day of weight measurement (P16), except on P3.

**FIGURE 2:**
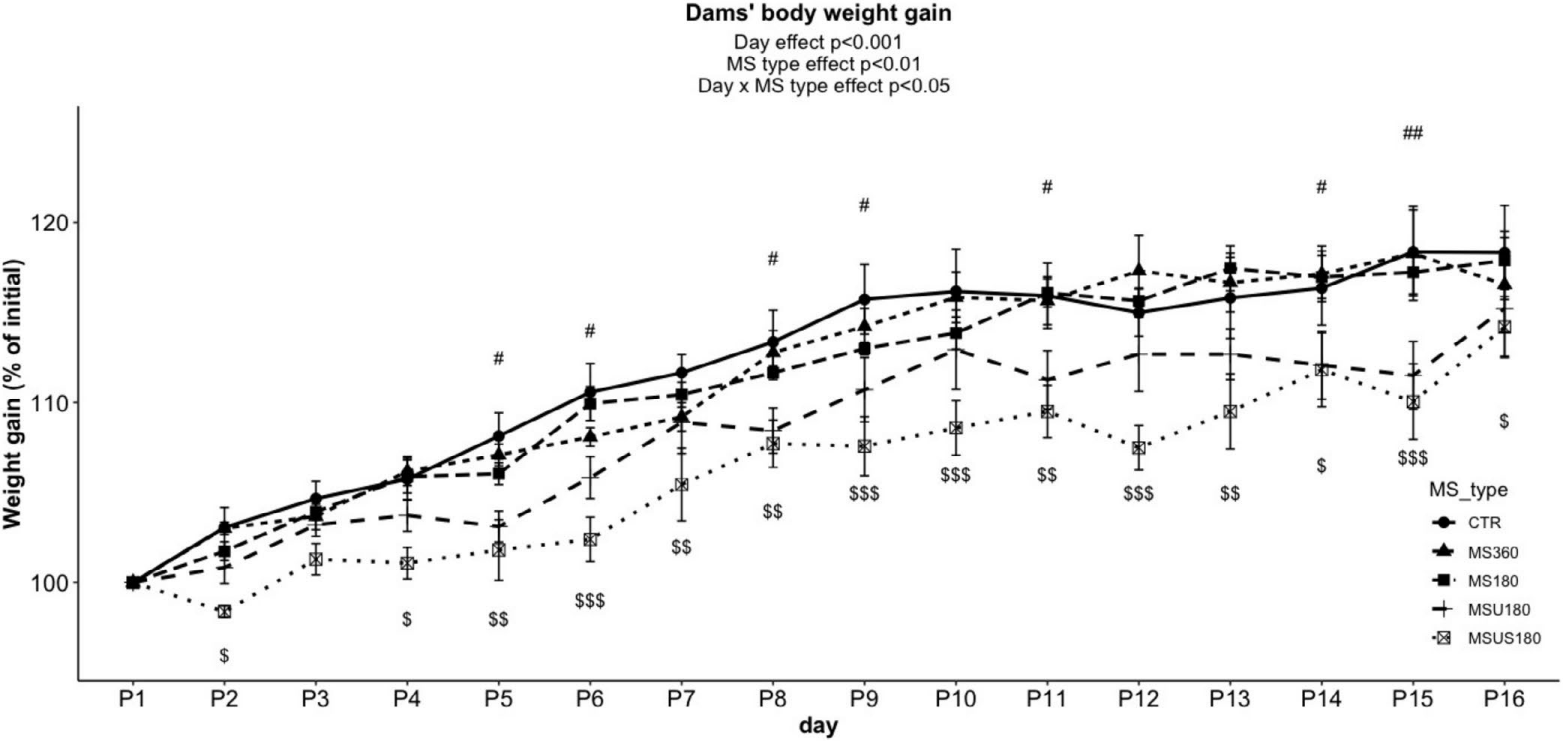
Effect of different maternal separation types on dams' body weight gain (mean ± SEM). MSU180 and MSUS180 dams gained significantly less weight than CTR dams. Significant differences on each day between MSU180 and CTR dams are indicated by #*p* < 0.05, ##*p* < 0.01. Significant differences on each day between MSUS180 and CTR dams are indicated by $*p* < 0.05, $$*p* < 0.01, $$$*p* < 0.001. CTR: Control handled pups; MS360: 6 h/day of predictable MS; MS180: 3 h/day of predictable MS; MSU180: 3 h/day of unpredictable MS; MSUS180: 3 h/day of unpredictable MS combined with maternal stress.

### 3.2 Maternal Care

The dams' nest occupancy during the first week was not significantly different between the MS types (Figure 3A; *F*_(4,38)_ = 0.70, *p* = 0.60). By contrast, a main effect of MS type during the second week was found (Figure 3B; *F*_(4,38)_ = 3.73, *p* < 0.05). Further analysis indicated a greater dams' nest occupancy of MSUS180 dams compared to CTR (*t* = 2.50, *p* < 0.05), MS360 (*t* = 3.45, *p* < 0.01), and MSU180 (*t* = 2.13, *p* < 0.05) dams; moreover, MS180 dams showed a greater nest occupancy than MS360 dams (*t* = 2.60, *p* < 0.05). No other pairwise comparisons were statistically significant.

**FIGURE 3:**
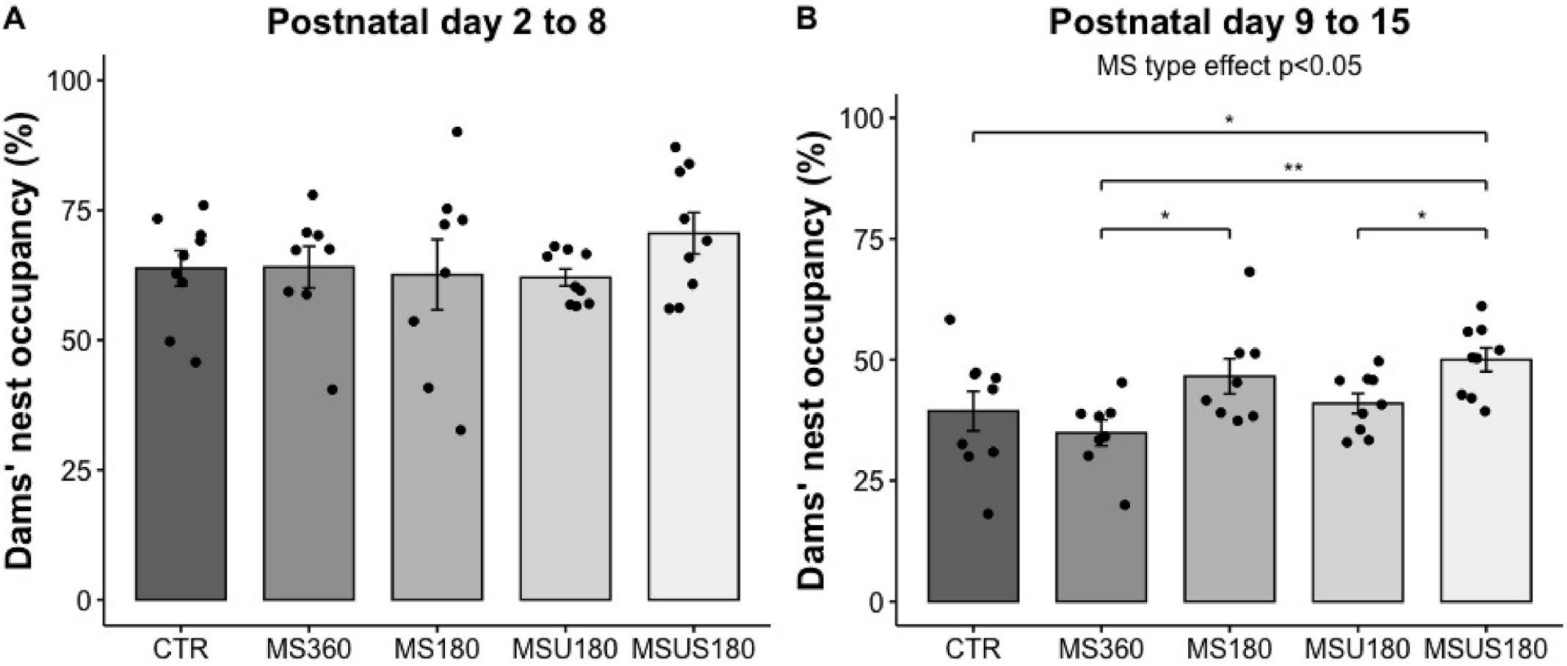
Dams' nest occupancy (mean ± SEM). (A) Postnatal week one (P2–P8). (B) Postnatal week two (P9–P15). *p < 0.05; **p < 0.01; ***p < 0.001. CTR, control handled animals; MS180, 3 h/day of predictable MS; MS360, 6 h/day of predictable MS; MSU180, 3 h/day of unpredictable MS; MSUS180, 3 h/day of unpredictable MS combined with maternal stress.

### 3.3 Offspring Growth

Two‐way ANOVAs were conducted separately for each postnatal day (P7, 14, 21, 28, 35, 42, 49, 56, 63, 70; mean ± sem per group/postnatal day in Table S2) to assess the effect of MS type, SERT genotype and their interaction on offspring growth, with pairwise comparisons included where appropriate (Table 1). On P7, further analysis following the main effect of MS type showed that offspring exposed to MS360, MS180 and MSU180 weighed more than CTR animals. On P21 and P28, a main effect of SERT genotype was found, with further analysis revealing that, in comparison to the wildtypes, SERT^+/−^ females weighed more, while SERT^−/−^ females weighed less. From P35 until the last measurement on P70, the main effect of SERT genotype persisted, with further analysis showing that SERT^−/−^ females weighed less than wildtype females.

**TABLE 1:** Outcomes of two‐way ANOVAs and pairwise comparisons following main/interaction effects of SERT genotype and MS type on offspring growth from P7–70.

Postnatal day	*n*	SERT genotype effect	MS type effect	SERT genotype × MS type effect	Pairwise comparisons versus SERT^+/+^	Pairwise comparisons versus CTR
P7	128	*F*_(2,113)_ = 2.21, *p* = 0.11	*F*_(4,113)_ = 4.00, *p* < 0.01**	*F*_(8,113)_ = 1.03, *p* = 0.42	NA	MS360 (*t* = 2.67, *p* < 0.01) MS180 (*t* = 2.67, *p* < 0.01) MSU180 (*t* = 2.65, *p* < 0.05)
P14	128	*F*_(2,113)_ = 1.33, *p* = 0.27	*F*_(4,113)_ = 2.92, *p* < 0.05*	*F*_(8,113)_ = 0.96, *p* = 0.47	NA	None significant
P21	125	*F*_(2,110)_ = 34.81, *p* < 0.001***	*F*_(4,110)_ = 3.89, *p* < 0.01**	*F*_(8,110)_ = 1.19, *p* = 0.31	SERT^+/−^ (t = 2.04, *p* < 0.05) SERT^−/−^ (*t* = −6.04, *p* < 0.001)	None significant
P28	125	*F*_(2,110)_ = 14.67, *p* < 0.001***	*F*_(4,110)_ = 1.55, *p* = 0.19	*F*_(8,110)_ = 1.08, *p* = 0.38	SERT^+/−^ (t = 2.38, *p* < 0.05) SERT^−/−^ (*t* = −3.19, *p* < 0.01)	N/A
P35	128	*F*_(2,113)_ = 18.05, *p* < 0.001***	*F*_(4,113)_ = 0.57, *p* = 0.69	*F*_(8,113)_ = 0.98, *p* = 0.46	SERT^−/−^ (*t* = −3.90, *p* < 0.001)	N/A
P42	127	*F*_(2,112)_ = 21.07, *p* < 0.001***	*F*_(4,112)_ = 0.30, *p* = 0.88	*F*_(8,112)_ = 1.18, *p* = 0.32	SERT^−/−^ (*t* = −7.79, *p* < 0.001)	N/A
P49	128	*F*_(2,113)_ = 17.34, *p* < 0.001***	*F*_(4,113)_ = 0.46, *p* = 0.77	*F*_(8,113)_ = 1.56, *p* = 0.13	SERT^−/−^ (*t* = −4.86, *p* < 0.001)	N/A
P56	128	*F*_(2,113)_ = 18.70, *p* < 0.001***	*F*_(4,113)_ = 0.36, *p* = 0.83	*F*_(8,113)_ = 1.46, *p* = 0.18	SERT^−/−^ (*t* = −4.36, *p* < 0.001)	N/A
P63	126	*F*_(2,111)_ = 14.55, *p* < 0.001***	*F*_(4,111)_ = 0.21, *p* = 0.93	*F*_(8,111)_ = 1.89, *p* = 0.07	SERT^−/−^ (*t* = −4.34, *p* < 0.001)	N/A
P70	128	*F*_(2,113)_ = 16.54, *p* < 0.001***	*F*_(4,113)_ = 0.32, *p* = 0.86	*F*_(8,113)_ = 1.57, *p* = 0.14	SERT^−/−^ (*t* = −3.99, *p* < 0.001)	N/A

*Note:* *p < 0.05; **p < 0.01; ***p < 0.001.

### 3.4 Behavioral Outcomes in Adult Offspring

#### 3.4.1 Open Field Test

Data from one MSUS180 SERT^−/−^ female were excluded due to technical issues during trial recording. Regarding the distance traveled in the OFT, a main effect of SERT genotype was found (Figure 4; *F*_(2,113)_ = 4.14, *p* < 0.05), in which SERT^−/−^ females traveled a greater distance than SERT^+/−^ females (t = 2.90, *p* < 0.01). Neither a main effect of MS type (*F*_(4,113)_ = 0.36, *p* = 0.84) nor a SERT genotype × MS type interaction effect (*F*_(8,113)_ = 0.63, *p* = 0.75) was found. Related to the frequency in the center area, no main/interaction effects were found (SERT genotype: *F*_(2,113)_ = 0.11, *p* = 0.90; MS type: *F*_(4,113)_ = 0.89, *p* = 0.47; SERT genotype × MS type: *F*_(8,113)_ = 0.94, *p* = 0.48, Figure S1A). Similarly, no main effect of SERT genotype (*F*_(2,113)_ = 0.86, *p* = 0.42), MS type (*F*_(4,113)_ = 0.96, *p* = 0.43), or their interaction (*F*_(8,113)_ = 1.23, *p* = 0.29) was found on the time that females spent in the center area (Figure S1B).

**FIGURE 4:**
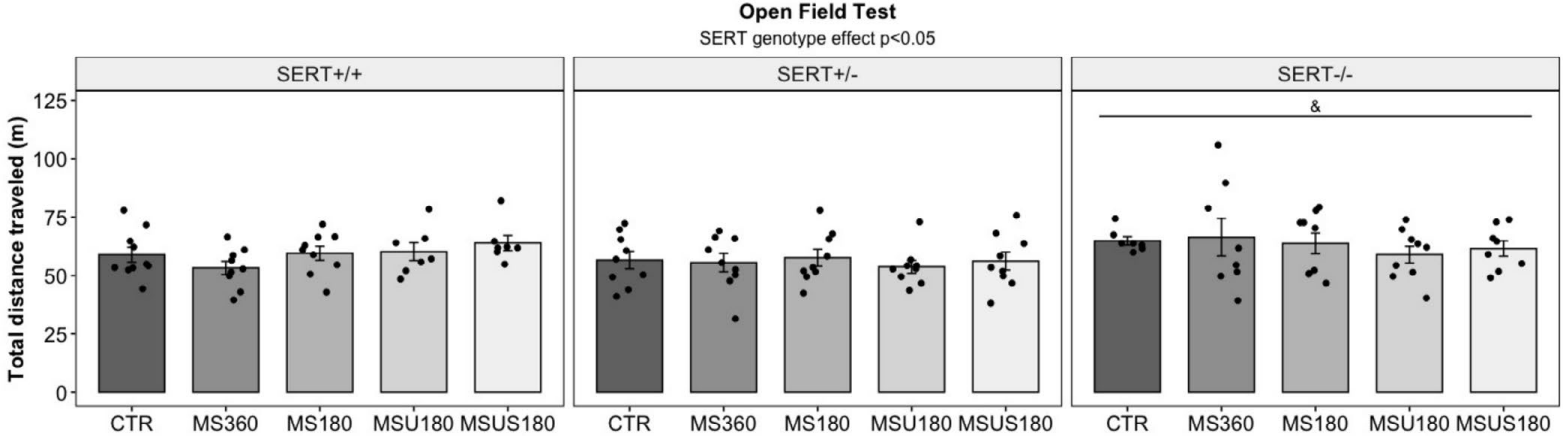
Distance traveled by control and maternally separated SERT^+/+^, SERT^+/−^ and SERT^−/−^ females in the OFT (mean ± SEM). &*p* < 0.05 versus SERT^+/−^. CTR, control handled animals; MS180, 3 h/day of predictable MS; MS360, 6 h/day of predictable MS; MSU180, 3 h/day of unpredictable MS; MSUS180, 3 h/day of unpredictable MS combined with maternal stress. Final sample sizes per group: SERT^+/+^ (CTR = 10; MS360 = 9; MS180 = 9; MSU180 = 7; MSUS180 = 7); SERT^+/−^ (CTR = 9; MS360 = 9; MS180 = 9; MSU180 = 9; MSUS180 = 9); SERT^−/−^ (CTR = 7; MS360 = 8; MS180 = 9; MSU180 = 9; MSUS180 = 8).

#### 3.4.2 Elevated Plus Maze

Data from one MSUS180 SERT^+/+^ female were excluded because only 1 min was recorded, and data from one CTR SERT^−/−^ female were missing; therefore, analyses included data from 127 females. A main effect of SERT genotype on the distance traveled (Figure 5A) was found (*F*_(2,112)_ = 9.13, *p* < 0.001), in which SERT^−/−^ females traveled significantly more distance than both SERT^+/+^ (*t* = 3.97, *p* < 0.001) and SERT^+/−^ (*t* = 3.20, *p* < 0.01) females. A main effect of MS type was also found (*F*_(4,112)_ = 2.93, *p* < 0.05), where MS360 (*t* = 2.26, *p* < 0.05) and MS180 (*t* = 2.49, *p* < 0.05) females traveled significantly less distance than the CTR group. The interaction effect of SERT genotype × MS type was not significant (*F*_(8,112)_ = 1.40, *p* = 0.20). Regarding the time that females spent in the open arms (Figure 5B), a main effect of SERT genotype was found (*F*_(2,112)_ = 11.42, *p* < 0.001), with SERT^−/−^ females spending significantly less time in the open arms than both SERT^+/+^ (*t* = 4.04, *p* < 0.001) and SERT^+/−^ (*t* = 4.40, *p* < 0.001) females. No main effect of MS type was found (*F*_(4,112)_ = 1.48, *p* = 0.21); however, a SERT genotype × MS type interaction effect was also found (*F*_(8,112)_ = 2.22, *p* < 0.05). Further analysis indicated that, within the SERT^+/+^ groups, females exposed to MS360 spent significantly less time in the open arms compared to the CTR (*t* = 3.31, *p* < 0.01), MS180 (*t* = 2.48, *p* < 0.05), and MSU180 (*t* = 2.10, *p* < 0.05) females. In addition, MS360 SERT^+/−^ females stayed for longer in the open arms than their wildtype counterparts (*t* = 2.15, *p* < 0.05). Regarding SERT^−/−^ females, those exposed to both MSU180 and MSUS180 spent significantly more time in the open arms compared to CTR SERT^−/−^ females (*t* = 2.06, *p* < 0.05; *t* = 2.08, *p* < 0.05, respectively); moreover, CTR SERT^−/−^ females spent significantly less time in the open arms than their wildtype (*t* = 4.20, *p* < 0.001) and heterozygous comparison groups (*t* = 2.89, *p* < 0.01); MS180 SERT^−/−^ females spent less time in the open arms than their wildtype (*t* = 3.18, *p* < 0.01) and heterozygous (*t* = 2.16, *p* < 0.05) counterparts; and the MSUS180 SERT^−/−^ group stayed less time in the open arms in comparison to their heterozygous comparison group (*t* = 2.40, *p* < 0.05).

**FIGURE 5:**
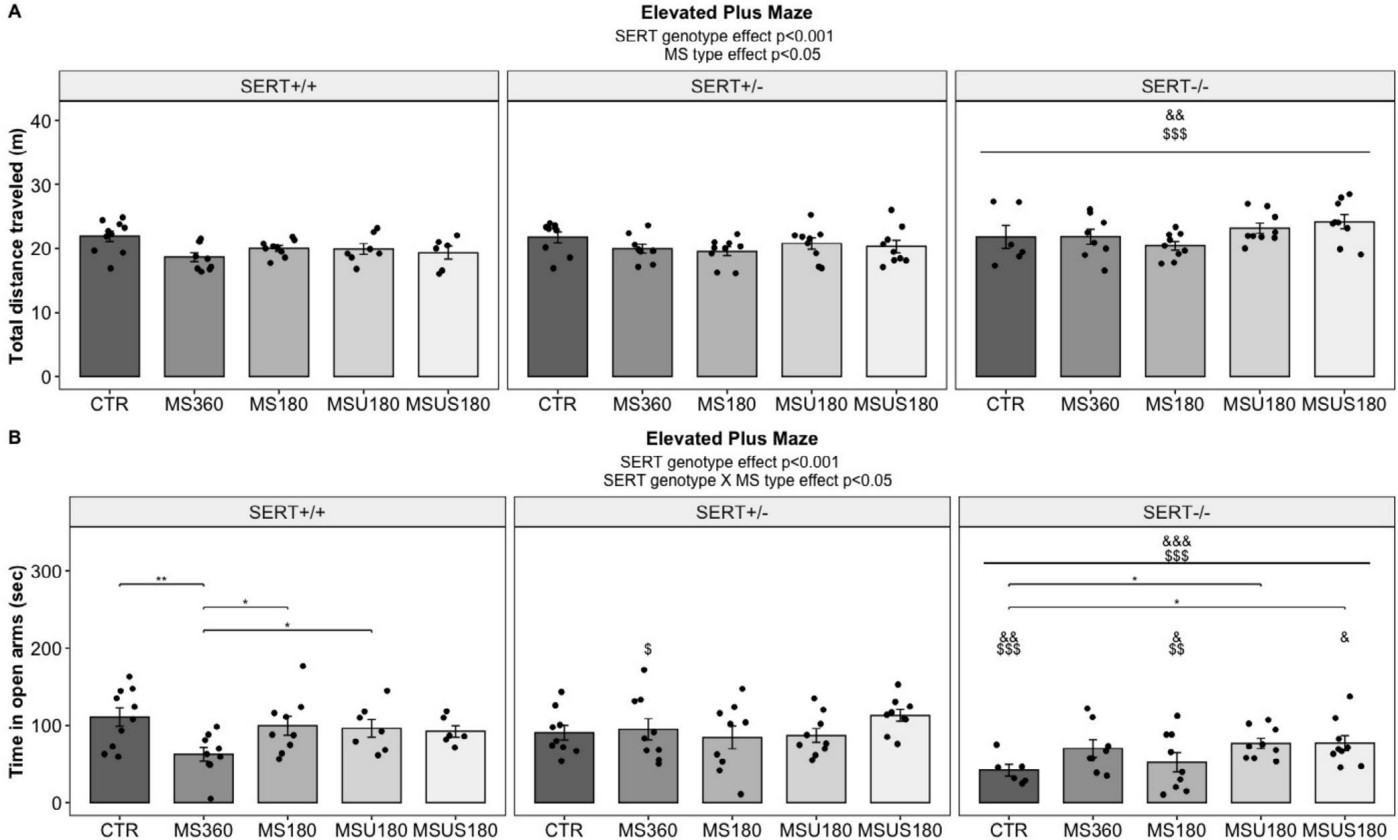
Outcomes of control and maternally separated SERT^+/+^, SERT^+/−^ and SERT^−/−^ females in the EPM (mean ± SEM). (A) Total distance moved. (B) Time spent in open arms. Symbols above solid long lines in SERT^−/−^ panels indicate significant pairwise comparisons following main effect of SERT genotype in two‐way ANOVAs. $*p* < 0.05; $$*p* < 0.01; $$$*p* < 0.001 versus SERT^+/+^; &*p* < 0.05; &&*p* < 0.01; &&&*p* < 0.001 versus SERT^+/−^; for differences between MS types: *p < 0.05; **p < 0.01. CTR, control handled animals; MS180, 3 h/day of predictable MS; MS360, 6 h/day of predictable MS; MSU180, 3 h/day of unpredictable MS; MSUS180, 3 h/day of unpredictable MS combined with maternal stress, Final sample sizes per group: SERT^+/+^ (CTR = 10; MS360 = 9; MS180 = 9; MSU180 = 7; MSUS180 = 6); SERT^+/−^ (CTR = 9; MS360 = 9; MS180 = 9; MSU180 = 9; MSUS180 = 9); SERT^−/−^ (CTR = 6; MS360 = 8; MS180 = 9; MSU180 = 9; MSUS180 = 9).

#### 3.4.3 Social Recognition

Females showing less than 5 s of social exploration during the sociability trial were excluded for this analysis. Data from one female were removed because one of the social stimuli escaped during the social recognition trial. One female did not explore the social stimulus during the sociability trial, another female did not explore any of the social stimuli during the social recognition trial, and another stayed in the same chamber during both trials, so she never knew about the new stranger. Therefore, data from 121 females were analyzed. A main effect of SERT genotype on the distance traveled during habituation was found (Figure 6A; *F*_(2,106)_ = 14.34, *p* < 0.001). SERT^−/−^ females traveled a greater distance than both SERT^+/+^ (*t* = 4.70, *p* < 0.001) and SERT^+/−^ (*t* = 4.52, *p* < 0.001) females. Neither a main effect of MS type (*F*_(4,106)_ = 1.33, *p* = 0.27) nor a SERT genotype × MS type interaction effect (*F*_(8,106)_ = 1.37, *p* = 0.22) was found. For the sociability index (Figure 6B), all groups preferred the social stimulus over the empty grid, as the index was above 0.5. A main effect of SERT genotype was found (*F*_(2,106)_ = 4.99, *p* < 0.01), with SERT^−/−^ females showing a higher preference for the social stimulus than SERT^+/+^ (*t* = 2.03, *p* < 0.05) and SERT^+/−^ (*t* = 3.12, *p* < 0.01) females. A trend of an MS type effect was also found (*F*_(4,106)_ = 2.36, *p* = 0.06), but pairwise comparisons did not reveal any significant difference. No SERT genotype × MS type interaction effect was found either (*F*_(8,106)_ = 0.58, *p* = 0.78). Regarding the duration of social exploration during the social recognition trial (Figure 6C), the SERT genotype effect was significant (*F*_(2,106)_ = 8.40, *p* < 0.001), with further analysis showing that SERT^−/−^ females displayed a higher amount of social exploration than both SERT^+/+^ (*t* = 3.81, *p* < 0.001) and SERT^+/−^ (*t* = 3.07, *p* < 0.01) females. No main effect of MS type (*F*_(4,106)_ = 0.77, *p* = 0.54) or SERT genotype × MS type interaction effect (*F*_(8,106)_ = 1.35, *p* = 0.23) was found. Regarding the recognition of the novel social stimulus, all groups exhibited a social recognition index above 0.5 except the CTR SERT^−/−^ group, in which the index was slightly below (0.48); however, no main effect of SERT genotype (*F*_(2,106)_ = 1.36, *p* = 0.26), MS type (*F*_(4,106)_ = 0.66, *p* = 0.62) or their interaction effect (*F*_(8,106)_ = 0.51, *p* = 0.85) was found (Figure 6D).

**FIGURE 6:**
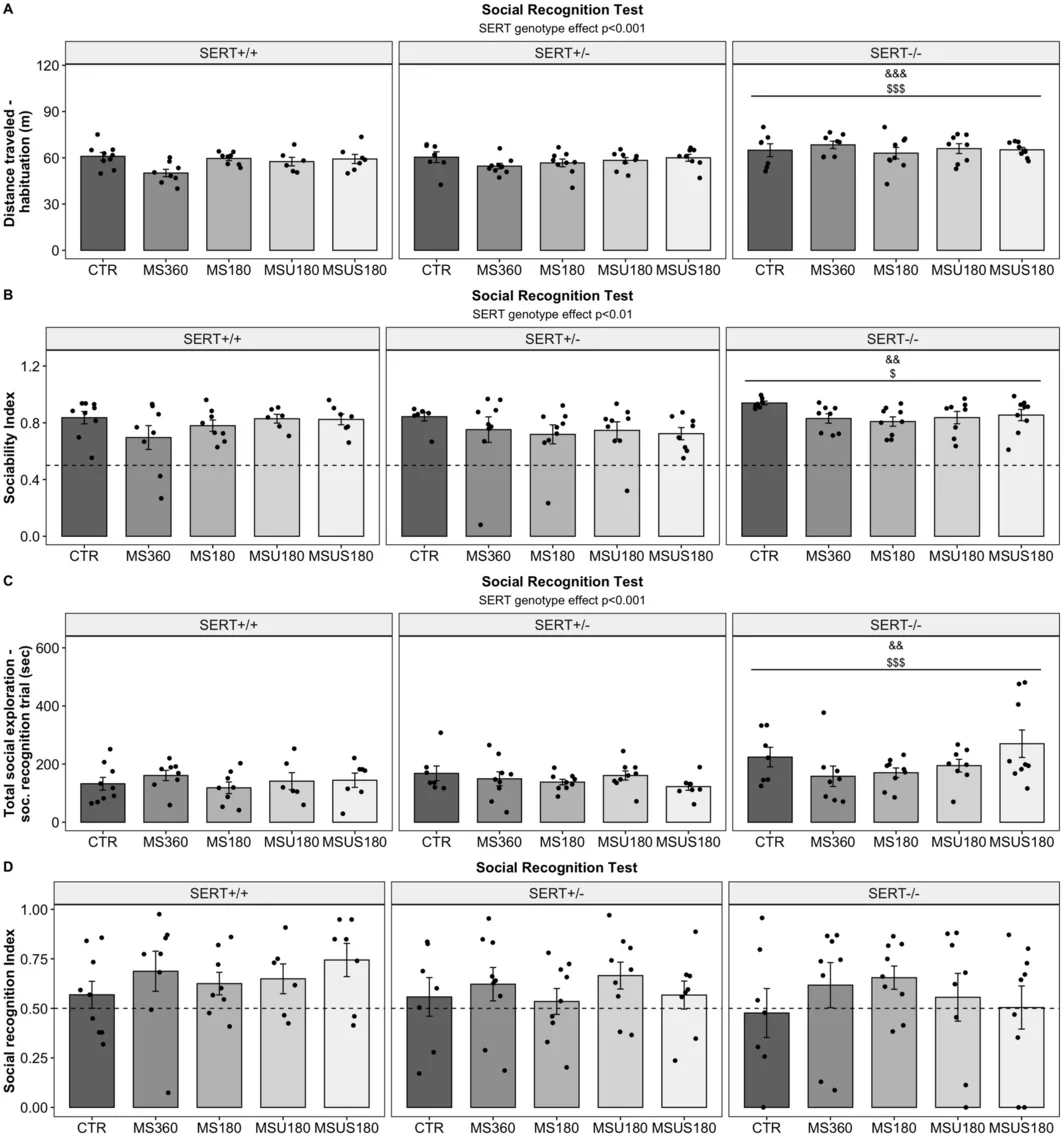
Outcomes of control and maternally separated SERT^+/+^, SERT^+/−^ and SERT^−/−^ females in the SR (mean ± SEM). (A) Total distance moved during habituation. (B) Sociability index in sociability trial. The dashed line at 0 indicates no preference. (C) Time spent with both social stimuli in the social recognition trial. (D) Social recognition index. Symbols above solid long lines in SERT^−/−^ panels indicate significant pairwise comparisons following main effect of SERT genotype in two‐way ANOVAs. $*p* < 0.05; $$$*p* < 0.001 versus SERT^+/+^; &&*p* < 0.01, &&&*p* < 0.001 versus SERT^+/−^. CTR, control handled animals; MS180, 3 h/day of predictable MS; MS360, 6 h/day of predictable MS; MSU180, 3 h/day of unpredictable MS; MSUS180, 3 h/day of unpredictable MS combined with maternal stress, Final sample sizes per group: SERT^+/+^ (CTR = 9; MS360 = 8; MS180 = 8; MSU180 = 6; MSUS180 = 7); SERT^+/−^ (CTR = 7; MS360 = 9; MS180 = 9; MSU180 = 9; MSUS180 = 8); SERT^−/−^ (CTR = 7; MS360 = 8; MS180 = 9; MSU180 = 8; MSUS180 = 9).

#### 3.4.4 Sucrose Preference Test

Due to leaking bottles or incorrect data entry of bottles' weight, some values per sucrose concentration were removed. Table S3 provides final sample sizes, group means, and SEMs for sucrose preference and sucrose intake, while Table 2 details the outcomes of the two‐way ANOVAs. No main effect of SERT genotype, MS type or their interaction effect was found on sucrose preference at 0.25%, 0.50% or 0.75% sucrose concentrations. A main effect of MS type was found on sucrose preference at the 1.0% concentration. Further analysis indicated that females exposed to MS360 had a higher sucrose preference than CTR group (*t* = 2.06, *p* < 0.05), while females exposed to MSUS180 showed a lower sucrose preference than those exposed to MS360 (*t* = 3.07, *p* < 0.01) and MSU180 (*t* = 2.05, *p* < 0.05). No main effect of SERT genotype or SERT genotype × MS type interaction effect was found. Regarding sucrose intake, no main effect of SERT genotype, MS type, or interaction effect was found at any of the sucrose concentrations.

**TABLE 2:** Outcomes of two‐way ANOVAs performed for the sucrose preference and sucrose intake per sucrose concentration.

Sucrose concentration	*n*	Sucrose preference			Sucrose intake		
		SERT genotype effect	MS type effect	SERT genotype × MS type effect	SERT genotype effect	MS type effect	SERT genotype × MS type effect
0.25%	123	*F*_(2,108)_ = 0.35, *p* = 0.71	*F*_(4,108)_ = 0.69, *p* = 0.60	*F*_(8,108)_ = 0.90, *p* = 0.52	*F*_(2,108)_ = 0.89, *p* = 0.42	*F*_(4,108)_ = 0.23, *p* = 0.92	*F*_(8,108)_ = 1.79, *p* = 0.09
0.50%	124	*F*_(2,109)_ = 1.50, *p* = 0.23	*F*_(4,109)_ = 1.52, *p* = 0.20	*F*_(8,109)_ = 0.62, *p* = 0.76	*F*_(2,109)_ = 1.09, *p* = 0.34	*F*_(4,109)_ = 0.78, *p* = 0.54	*F*_(8,109)_ = 1.25, *p* = 0.28
0.75%	124	*F*_(2,109)_ = 0.13, *p* = 0.88	*F*_(4,109)_ = 0.49, *p* = 0.74	*F*_(8,109)_ = 1.20, *p* = 0.30	*F*_(2,109)_ = 2.27, *p* = 0.11	*F*_(4,109)_ = 0.40, *p* = 0.81	*F*_(8,109)_ = 0.86, *p* = 0.55
1.00%	120	*F*_(2,105)_ = 0.14, *p* = 0.87	*F*_(4,105)_ = 2.52, *p* < 0.05*	*F*_(8,105)_ = 1.08, *p* = 0.38	*F*_(2,107)_ = 0.49, *p* = 0.61	*F*_(4,107)_ = 1.40, *p* = 0.24	*F*_(8,107)_ = 0.60, *p* = 0.78

*Note:* *p < 0.05.

#### 3.4.5 Home Cage Emergence

For the latency to escape the home cage in the HCE test (Figure 7), a main effect of SERT genotype was found (*F*_(2,114)_ = 8.84, *p* < 0.001), with further analysis indicating that SERT^−/−^ females took significantly more time to escape the home cage than both SERT^+/+^ (*t* = 3.17, *p* < 0.01) and SERT^+/−^ (*t* = 4.06, *p* < 0.001) females. No main effect of MS type (*F*_(4,114)_ = 1.11, *p* = 0.36), or SERT genotype × MS type interaction effect (*F*_(8,114)_ = 1.76, *p* = 0.09) was found.

**FIGURE 7:**
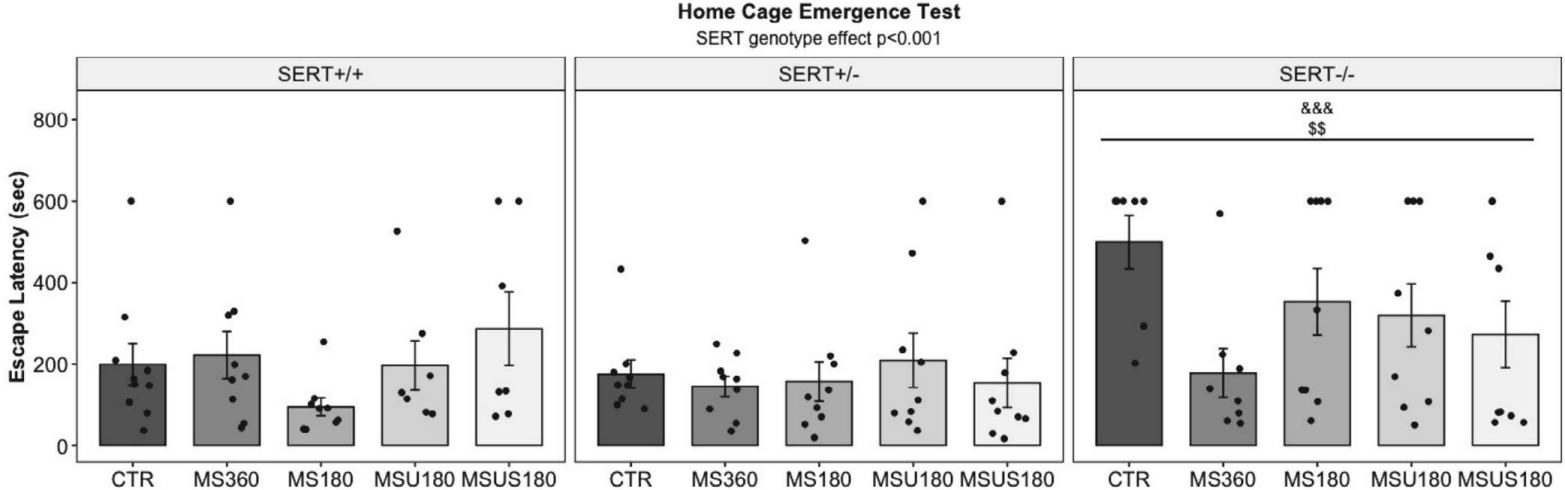
Latency to escape from the home cage in control and maternally separated SERT^+/+^, SERT^+/−^ and SERT^−/−^ females in the HCE test (mean ± SEM). Symbols above the solid long line in the SERT^−/−^ panel indicate significant pairwise comparisons following main effect of SERT genotype in the two‐way ANOVA. $$*p* < 0.01 versus SERT^+/+^; &&&*p* < 0.001 versus SERT^+/−^. CTR, control handled animals; MS180, 3 h/day of predictable MS; MS360, 6 h/day of predictable MS; MSU180, 3 h/day of unpredictable MS; MSUS180, 3 h/day of unpredictable MS combined with maternal stress.

#### 3.4.6 Novel Object Recognition

Data from two MS180 SERT^−/−^ females were excluded because of missing data. Females that did not explore any of the objects, or explored them for less than 5 s, were also excluded. Removing these data, 116 females composed the final sample size. Regarding the DI of the novel object (Figure 8), one‐sided paired *t*‐test comparisons between the novel object DI of each group and the DI_fic_ indicated that all females discriminated the novel object significantly above zero (Table 3). No main effect of SERT genotype (*F*_(2,101)_ = 1.11, *p* = 0.33) or MS type (*F*_(4,101)_ = 1.20, *p* = 0.32) was found; however, two‐way ANOVA revealed a SERT genotype × MS type interaction effect (*F*_(8,101)_ = 2.44, *p* < 0.05), where the effect of MSU180 was SERT‐genotype‐specific. SERT^+/+^ females exposed to MSU180 showed a lower DI than SERT^+/+^ females exposed to MS180 (*t* = 2.50, *p* < 0.05); moreover, SERT^+/−^ females exposed to MSU180 had a higher DI than both their wildtype (*t* = 2.60, *p* < 0.05) and homozygous (*t* = 3.70, *p* < 0.001) counterparts, and than SERT^+/−^ females exposed to MS360 (*t* = 2.11, *p* < 0.05). In addition, SERT^−/−^ females exposed to MSU180 showed a lower DI than the CTR (*t* = 2.81, *p* < 0.01), MS360 (*t* = 2.84, *p* < 0.01) and MSUS180 (*t* = 2.31, *p* < 0.05) SERT^−/−^ females.

**FIGURE 8:**
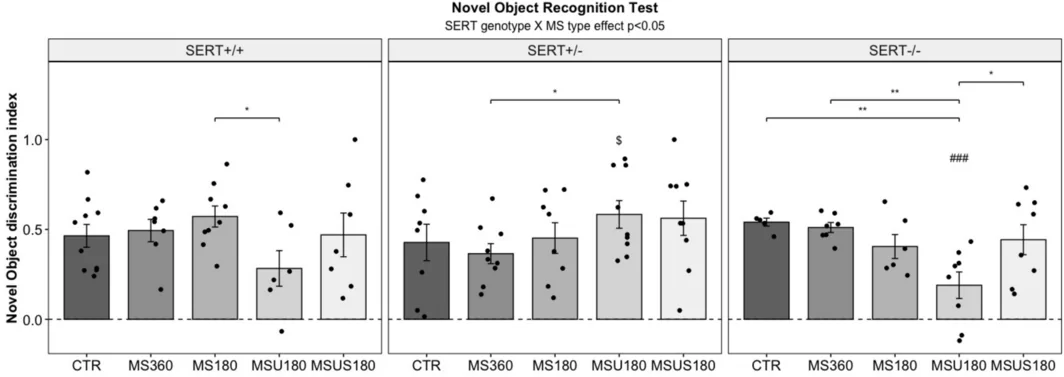
Discrimination index of the novel object in control and maternally separated SERT^+/+^, SERT^+/−^, and SERT^−/−^ females (mean ± SEM). The dashed line indicates no discrimination (equal exploration) between objects. Each group was compared with its respective fictional comparison group by running one‐sided paired t‐test comparisons. (See Table 3 in the main text for all paired t‐test comparisons). $*p* < 0.05 versus SERT+/+; ###*p* < 0.001 versus SERT+/−; for differences between MS types: *p < 0.05; **p < 0.01. CTR, control handled animals; MS180, 3 h/day of predictable MS; MS360, 6 h/day of predictable MS; MSU180, 3 h/day of unpredictable MS; MSUS180, 3 h/day of unpredictable MS combined with maternal stress. Final sample sizes per group: SERT^+/+^ (CTR = 10; MS360 = 7; MS180 = 9; MSU180 = 6; MSUS180 = 7); SERT^+/−^ (CTR = 8; MS360 = 9; MS180 = 8; MSU180 = 9; MSUS180 = 9); SERT^−/−^ (CTR = 5; MS360 = 7; MS180 = 6; MSU180 = 8; MSUS180 = 8).

**TABLE 3:** Outcomes of one‐sided paired *t*‐test comparisons between the novel object DI of each group and the DI_fic_ of its respective fictional comparison group.

SERT genotype	MS type	DI_fic_			Novel object DI			One‐sided paired *t*‐test	
		Mean	SD	±SEM	Mean	SD	±SEM	Statistic	*p*
SERT^+/+^	CTR	0	0.22	0.07	0.47	0.20	0.06	*t*_(9)_ = 5.55	< 0.001***
	MS360	0	0.17	0.06	0.49	0.17	0.06	*t*_(6)_ = 4.47	< 0.01**
	MS180	0	0.31	0.10	0.57	0.18	0.06	*t*_(8)_ = 5.77	< 0.001***
	MSU180	0	0.19	0.08	0.28	0.24	0.10	*t*_(5)_ = 2.22	< 0.05*
	MSUS180	0	0.43	0.16	0.47	0.32	0.12	*t*_(6)_ = 2.64	< 0.05*
SERT^+/−^	CTR	0	0.15	0.05	0.43	0.29	0.10	*t*_(7)_ = 3.77	< 0.01**
	MS360	0	0.17	0.06	0.37	0.17	0.06	*t*_(8)_ = 5.98	< 0.001***
	MS180	0	0.24	0.09	0.45	0.24	0.09	*t*_(7)_ = 3.45	< 0.01**
	MSU180	0	0.31	0.10	0.58	0.23	0.08	*t*_(8)_ = 4.35	< 0.01**
	MSUS180	0	0.28	0.09	0.56	0.29	0.10	*t*_(8)_ = 3.56	< 0.01**
SERT^−/−^	CTR	0	0.20	0.09	0.54	0.05	0.02	*t*_(4)_ = 6.08	< 0.01**
	MS360	0	0.25	0.09	0.51	0.07	0.03	*t*_(6)_ = 5.16	< 0.01**
	MS180	0	0.18	0.07	0.41	0.16	0.07	*t*_(5)_ = 3.48	< 0.01**
	MSU180	0	0.38	0.14	0.19	0.21	0.07	*t*_(7)_ = 1.86	0.05
	MSUS180	0	0.18	0.06	0.44	0.24	0.08	*t*_(7)_ = 6.48	< 0.001***

*Note:* *p < 0.05; **p < 0.01; ***p < 0.001.

#### 3.4.7 Forced Swim Test

Two SERT^−/−^ females unfortunately died despite intervention during habituation, therefore, data from 127 females were analyzed. A main effect of SERT genotype on the percentage of immobility was found (Figure 9; *F*_(2,112)_ = 31.24, *p* < 0.001), with SERT^−/−^ females showing a higher percentage of immobility compared to both SERT^+/+^ (*t* = 6.49, *p* < 0.001) and SERT^+/−^ (*t* = 7.23, *p* < 0.001) females. No main effect of MS type was found (*F*_(4,112)_ = 1.43, *p* = 0.23), however, a SERT genotype × MS type interaction effect was found (*F*_(8,112)_ = 2.03, *p* < 0.05). MSU180 SERT^+/+^ females showed greater immobility than the CTR SERT^+/+^ females (*t* = 2.08, *p* < 0.05). The percentage of immobility in MS360 SERT^+/−^ females was lower than the CTR (*t* = 2.48, *p* < 0.05), MSU180 (*t* = 3.60, *p* < 0.001), and MSUS180 (*t* = 1.98, *p* < 0.05) SERT^+/−^ females. The percentage of immobility was also higher in CTR SERT^−/−^ females compared to their SERT counterparts (vs. SERT^+/+^ *t* = 3.39, *p* < 0.001; vs. SERT^+/−^ *t* = 2.25, *p* < 0.05); moreover, the percentage of immobility in MS360 SERT^−/−^ females was higher in comparison to both SERT^+/+^ (*t* = 3.23, *p* < 0.01) and SERT^+/−^ (*t* = 5.47, *p* < 0.001) MS360 females; similarly, MS180 SERT^−/−^ females showed a higher percentage of immobility than their SERT counterparts (vs. SERT^+/+^ *t* = 4.62, *p* < 0.001, vs. SERT^+/−^ *t* = 4.21, *p* < 0.001); and the same was found by comparing MSUS180 SERT^−/−^ females with the MSUS180 SERT^+/+^ *t* = 2.02, *p* < 0.05, and MSUS180 SERT^+/−^ *t* = 2.79, *p* < 0.01 females.

**FIGURE 9:**
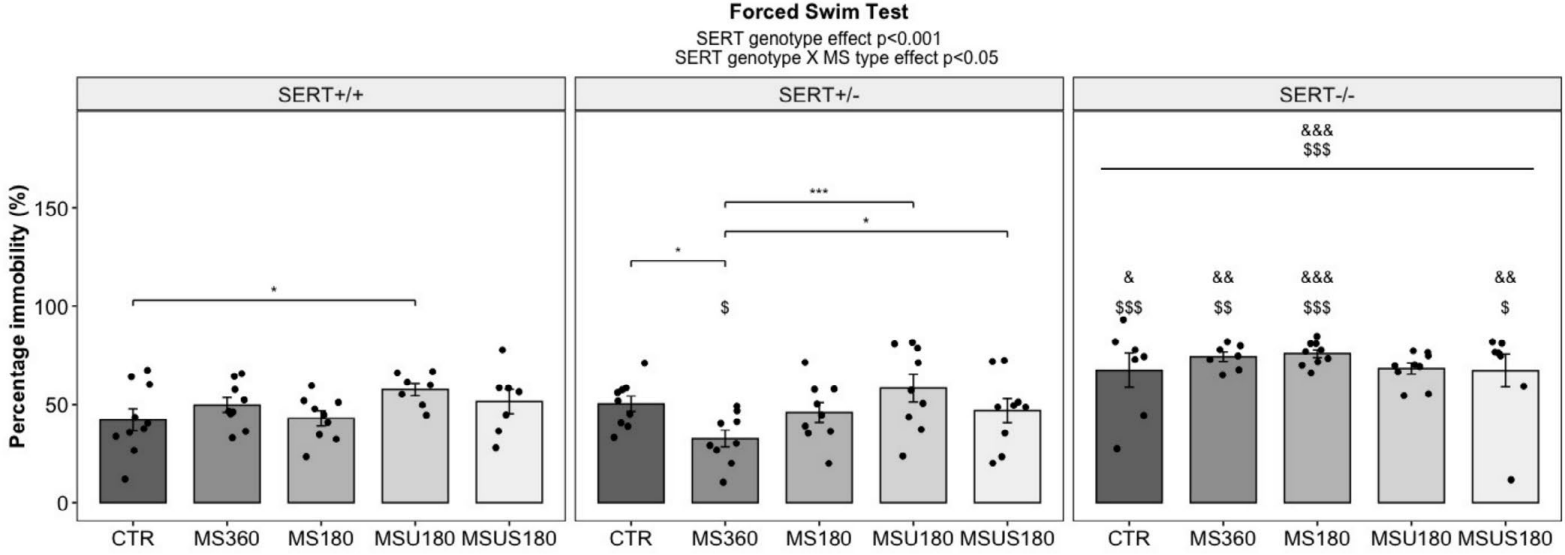
Percentage immobility during the forced swimming in control and maternally separated SERT^+/+^, SERT^+/−^ and SERT^−/−^ females (mean ± SEM). Symbols above the solid long line in the SERT^−/−^ panel indicate significant pairwise comparisons following main effect of SERT genotype in the two‐way ANOVA. $*p* < 0.05; $$*p* < 0.01; $$$*p* < 0.001 versus SERT+/+; &*p* < 0.05; &&*p* < 0.01; &&&*p* < 0.001 versus SERT+/−; for differences between MS groups: *p < 0.05; ***p < 0.001. CTR, control handled animals; MS180, 3 h/day of predictable MS; MS360, 6 h/day of predictable MS; MSU180, 3 h/day of unpredictable MS; MSUS180, 3 h/day of unpredictable MS combined with maternal stress. Final sample sizes per group: SERT^+/+^ (CTR = 10; MS360 = 9; MS180 = 9; MSU180 = 7; MSUS180 = 7); SERT^+/−^ (CTR = 9; MS360 = 9; MS180 = 9; MSU180 = 9; MSUS180 = 9); SERT^−/−^ (CTR = 7; MS360 = 7; MS180 = 9; MSU180 = 9; MSUS180 = 8).

## 4 Discussion

We examined whether SERT genotype interacted with MS as an early life stressor in the development of depression‐ and anxiety‐like behaviors in adult female rats, and whether these effects depended on the duration, predictability, and maternal stress associated with MS. Several behavioral tests were used to assess affective, cognitive, and social behaviors. SERT^−/−^ females exhibited passive stress coping, higher anxiety‐like responses, and increased social exploration together with increased locomotor activity, indicating a robust genotype‐driven phenotype. Predictable MS (i.e., MS180 and MS360) induced limited anxiety‐like responses, whereas prolonged predictable separation (MS360) was associated with increased hedonic responding. SERT genotype × MS interactions revealed behavioral outcomes that varied depending on both genotype and the type of MS exposure. Unpredictable MS paradigms (MSU180 and MSUS180) attenuated some anxiety‐related measures in SERT^−/−^ females. In the cognitive domain, MSU180 differentially influenced object recognition, improving performance in SERT^+/−^ females while reducing it in SERT^−/−^ females. Lastly, SERT^+/−^ females exhibited less passive stress coping when exposed to MS360.

### 4.1 Maternal and Offspring Body Weight Effects

We found that MSU180 and MSUS180 dams gained less weight, which was particularly pronounced in the MSUS180 group. This suggests that unpredictability combined with direct stress to the dam altered her metabolic regulation, known to be highly sensitive to repeated stress exposure [54]. Reflecting this maternal metabolic disruption, MSUS180 offspring did not exhibit the transient increase in body weight at P7 observed in the other MS groups. While this P7 increase in the other MS groups might reflect a compensatory surge in milk intake upon reunion, we speculate it was absent in the MSUS180 group due to a stress‐induced reduction of lactation, which has been reported in dams under stress during the lactating period [55]. Furthermore, the body weight reduction in SERT^−/−^ females from weaning through adulthood represents a genotype‐driven effect that has been previously observed in SERT^−/−^ rodents when compared to wildtypes [56].

### 4.2 SERT Genotype Effects

In both the EPM and HCE tasks, SERT^−/−^ females stayed for longer in enclosed, more protected spaces and consistently traveled longer distances in the EPM, the SR, and during the first 5 min of the OFT (data not shown). Although center‐area exploration in the OFT did not differ between genotypes, the enhanced avoidance of open spaces observed in the EPM and the HCE test, alongside increased locomotion, is consistent with the anxiety‐like phenotype that has been previously reported for this genotype [22, 57, 58]. The lifelong absence of SERT expression may predispose these animals to heightened aversive responses to novel environments, potentially manifesting as increased escape behaviors when confronted with proximal or highly salient threats [59].

We found that sociability did not differ between SERT^+/−^ and SERT^+/+^ females, in line with previous findings that showed no difference in social behaviors, including social exploration, approach, and play [60]. By contrast, SERT^−/−^ females showed increased sociability compared to wildtypes and SERT^+/−^ females. While this finding diverges from reports showing reduced social preference in female SERT knockout mice [58] or reduced social contact in male SERT knockout rats [61], these discrepancies may reflect sex‐ or species‐dependent differences as well as methodological factors. Importantly, both Tanaka et al. [58] and Golebiowska et al. [61] evaluated social behavior using absolute exploration time, a measure that does not account for individual exploratory differences. In contrast, relative measures such as the sociability index used in our study correct for the total time spent exploring both stimuli, making the final index independent of an animal's baseline exploration levels [53]. Consequently, it cannot be ruled out that their findings of reduced sociability, measured by absolute duration, may be due to differences in exploratory behavior rather than in the social stimulus preference. We consider that the sociability index reported here may account for the motivation of females to investigate an unfamiliar conspecific, independently of exploration levels. From this view, SERT^+/−^ females may have maintained unaltered social motivation, whereas SERT^−/−^ females showed enhanced motivation. Although we found no differences in social recognition index between the SERT genotypes, SERT^−/−^ females spent more time exploring the two social stimuli than both wildtype and SERT^+/−^ females, further supporting the notion of differences in exploratory behavior of SERT^−/−^ females towards social stimuli with no effect on the discrimination of the novel social stimulus.

Consistent with prior work, SERT^−/−^ females displayed increased immobility in the FST [62, 63, 64], suggesting that a lifetime lack of SERT expression predisposes SERT^−/−^ females to exhibit a passive stress coping style when they face a stressful situation. It is worth noting that a more extreme presentation of this phenotype was actually observed in the two SERT^−/−^ females that sank during the induction phase; because these highly animals could not complete the test and were therefore excluded from the final analysis, the immobility observed in the remaining SERT^−/−^ females likely represent a conservative estimate of this passive stress coping phenotype associated with SERT deficiency. While such a conservation‐withdrawal response reflects a conserved behavioral adaptation to stress [65], its sensitivity to antidepressants supports the assessment of a passive stress coping response as a measure of vulnerability to stress‐induced, depressive‐like behaviors [66]. In fact, successful breeding to produce genetically susceptible animals to a depressive‐like phenotype has been based on the selection of animals with large reduction of mobility in the FST [67]. Therefore, the robust immobility phenotype observed in SERT^−/−^ females reinforces the validity of this model for studying the underlying neurobiology of stress vulnerability and depressive‐like states.

We did not observe anhedonia‐like responses in our SERT+/− or SERT−/− females compared to wildtypes across the sucrose concentrations tested (0.25%–1%). Previous findings in SERT knockout rodents have been mixed. For instance, Schraut et al. [68] compared wildtype and SERT^+/−^ female mice and found no difference in sucrose preference between the groups. Similarly, no reduced sucrose preference was observed when comparing SERT^+/−^ and SERT^−/−^ with wildtype male mice [69]. Sucrose consumption did also not differ between wildtype and SERT knockout mice when tested at low (0.7%), medium (7.0%), or high (34%) concentrations of sucrose [70]. By contrast, Shoji et al. [64] found a reduced sucrose preference in both SERT^+/−^ and SERT^−/−^ compared to wildtype male mice using a 1% sucrose solution. When measured in rats, a lower sucrose preference was found in SERT^−/−^ males compared to wildtypes [71], whereas another study reported lower sucrose preference in SERT^−/−^ females but not in SERT^−/−^ males [63]. Notably, lower sucrose preferences in SERT^−/−^ compared to wildtype animals have primarily been observed at moderate to high concentrations, at 3% [71], 8% [72], and 10% [63], but not at 2% [63]. We administered only low concentrations (0.25%–1%), which may explain the absence of genotype effects. In fact, all females discriminated sucrose from water even at the lowest concentration, suggesting intact reward sensitivity across genotypes. It is possible that SERT^−/−^ females exhibit a blunted increase in hedonic responding at higher rewarding magnitudes, as observed by Olivier, Van Der Hart et al. [63]. Our SERT^−/−^ females did show a gradual increase in their hedonic response similar to wildtypes when sucrose concentrations were very low (data not shown); whether the reward magnitude remains as in wildtypes at higher doses remains to be established.

Altogether, SERT^−/−^ females exhibited a behavioral profile characterized by anxiety‐ and depressive‐like responses, supporting the utility of this model for investigating serotonergic mechanisms underlying neuropsychiatric symptoms in females. We wonder whether the disruption of serotonergic signaling during development may contribute to long‐lasting changes in neural circuits involved in emotional regulation. Chronic extracellular 5‐HT elevation by disrupted SERT function during sensitive windows of development can downregulate 5‐HT receptor expression [73, 74, 75], alter the excitatory/inhibitory balance [76], and reduce synaptic BDNF [77]. We hypothesize that these changes might collectively enhance amygdala reactivity and weaken prefrontal control, contributing to the behavioral phenotype in SERT^−/−^ females. Moreover, interactions with ovarian hormones might further shape the female‐specific phenotype, as estrogen interacts with serotonergic transmission by modulating 5‐HT receptors' expression and SERT activity [78, 79, 80], and influences oxytocinergic/vasopressinergic signaling in the nucleus accumbens and the amygdala [81, 82]. Elucidating how developmental serotonergic alterations interact with ovarian hormones to shape circuit‐level and behavioral outcomes represents an important direction for understanding sex‐specific vulnerability to neuropsychiatric disorders.

### 4.3 Maternal Separation Type Effects

Two significant findings were observed. First, females exposed to MS180 and MS360 exhibited reduced locomotor activity in the EPM. While reduced locomotion may reflect altered anxiety‐like behavior, it could equally reflect heightened defensive or risk‐assessment behaviors such as freezing or stretch‐attend postures in response to novelty [83]. Indeed, prior ELS studies, predominantly in male rodents, have linked MS to elevated rearing, stretching, or head‐dipping responses [84, 85, 86]. However, because only total time spent in the open arm was quantified, we could not distinguish active exploration from periods of immobility within the open arms. Moreover, ethological measures such as freezing, head dipping, and stretch‐attend postures were not quantified. Therefore, this interpretation remains speculative. Future work incorporating these measures may be necessary to determine whether reduced locomotion in MS females represents fear responses or distinct exploratory strategies.

Second, females exposed to MS360, but not to other MS protocols, showed a higher hedonic response, reflected by their preference for 1.0% sucrose solution. This pattern may reflect a threshold‐dependent effect, where a longer daily separation duration (6 h vs. 3 h) under predictable conditions is required to alter adult reward sensitivity in females. Alternatively, because this sucrose preference was observed selectively at a single sucrose concentration within one experimental group, the possibility that this represents an isolated finding must also be considered. Although previous studies have suggested that MS may influence incentive processing rather than primary hedonic responding [87], the present study was not designed to distinguish between these processes. Consequently, any interpretation involving altered reward‐based learning processes remains speculative. Whether prolonged predictable MS (MS360) alters reward sensitivity in females requires further validation using additional reward paradigms, a broader range of sucrose concentrations, and independent cohorts.

Overall, MS did not consistently induce negative outcomes across behavioral domains in female rats. Instead, its effects were task‐specific and dependent on MS type. Thus, predictability and duration of MS critically determine behavioral outcomes and do not inevitably result in long‐term deficits in females.

### 4.4 SERT Genotype × MS Type Interaction Effects

Across behavioral tests, we observed multiple interaction effects between SERT genotype and MS type, in which females responded differently to MS type depending on their SERT genotype. First, while SERT^−/−^ control females spent less time in the open arms compared to the other genotypes, SERT^−/−^ females exposed to either type of unpredictable MS spent a longer duration in the open arms, thus suggesting that the unpredictability of MS may buffer the heightened anxiety phenotype observed in SERT^−/−^ females. We speculate that MS unpredictability may have influenced maternal caregiving, thereby reducing anxiety‐like behaviors in SERT^−/−^ female offspring. In support of this idea, enhanced maternal care was associated with reduced anxiety in offspring [88, 89], whereas low maternal care increased it [90, 91]. What is more, Carola et al. [92] demonstrated that SERT^+/−^, but not wildtype offspring exhibited increased anxiety‐like responses when exposed to low maternal care, suggesting that serotonergic function may heighten susceptibility to caregiving‐related environmental modulation. Although we were unable to quantify pup‐directed maternal behaviors due to dome‐shaped nest structures, it is worth noting that MSUS180 dams showed a higher occupancy of the nest during the second postnatal week. This observation supports the possibility that unpredictable MS combined with maternal stress may promote compensatory caregiving behaviors that buffer anxiety in SERT^−/−^ females. Whether SERT^−/−^ offspring are particularly sensitive to enhanced maternal care remains an important question for future studies. If future studies confirm a mediating role for maternal caregiving, this would further support the concept that early caregiving quality can shape resilience to environmental stress in genetically vulnerable individuals.

Another interaction effect emerged in which SERT^+/−^ females exposed to unpredictable MS (i.e., MSU180) exhibited higher object recognition than the other genotypes, and SERT^−/−^ females exposed to the same MS showed lower object recognition than females exposed to other MS types (except MS180). When tested 1–4 h after training [93, 94, 95], object recognition is generally preserved across SERT genotypes, with SERT^+/−^ males and females outperforming wildtypes at a 3 h‐delay, but not at 4 h [95]. By contrast, both SERT^+/−^ and SERT^−/−^ males [93] and SERT^−/−^ females [96] failed to discriminate the novel object when the delay was 8 h. Here, we tested the animals after only a 45‐s retention interval, which likely preserved object recognition in all groups. Because the retention interval was only 45 s, our findings should primarily be interpreted as reflecting immediate object recognition rather than recognition memory over longer retention intervals. Although very short retention intervals have previously been shown to support reliable object discrimination in rats [50, 97], they place relatively limited demands on memory retention compared with the longer delays commonly used in standard NOR paradigms. That said, the higher recognition of SERT^+/−^ but lower recognition in SERT^−/−^ females when exposed to MSU180 is worth noting and may indicate that sensitivity to unpredictable MS differs between SERT variants. Although the present task primarily assessed immediate object recognition, underlying genotype‐dependent differences in hippocampal function may still have contributed to the observed behavioral differences. We speculate that the distinct serotonergic system in each genotype influences how MSU180 affects brain regions involved in memory processes. Although object recognition involves hippocampal processing [98], the short retention interval used here likely imposed relatively limited demands on memory retention. In SERT^−/−^, but not SERT^+/−^ rodents, the lifelong absence of SERT leads to presynaptic and neurochemical adaptations in the hippocampus [23, 63], which we propose may make this region vulnerable to stress‐induced dysregulation. In addition, MS disrupts the hypothalamic–pituitary–adrenal (HPA) axis and alters the molecular and electrophysiological properties of the hippocampus [99, 100]. The combination of altered serotonergic signaling and stress‐induced hippocampal vulnerability may therefore account for the reduced object recognition of MSU180 SERT^−/−^ females. The mechanisms by which MSU180 enhanced recognition memory in SERT^+/−^ females remain unclear, but might reflect an optimal level of environmental challenge that increases cognitive performance in moderately vulnerable animals.

Notably, MSUS180 did not impair object recognition in SERT^−/−^ females, despite its unpredictable nature. Given that MSUS180 dams exhibited greater nest occupancy, this finding raises the question of whether enhanced maternal care may have occurred during this period of nest occupancy to mitigate the stress‐related cognitive impairments in these animals. The lack of significant memory outcomes following predictable MS (MS180 and MS360) further supports the notion that unpredictability, rather than separation per se, may be critical for eliciting adverse cognitive effects in SERT^−/−^ females. Nevertheless, it remains to be demonstrated whether MSUS180 dams actually provided higher levels of maternal care, as direct behavioral tracking was precluded by the reduced visibility of the nest.

Finally, a SERT genotype × MS interaction was observed in the FST, with reduced immobility in SERT^+/−^ females exposed to MS360. While this could reflect a less passive coping style in response to stress [65], it may also indicate heightened arousal or agitation in an aversive context, resembling a panic‐like response [59]. Further studies examining amygdala reactivity, prefrontal control, and midbrain function in SERT^+/−^ females under prolonged MS exposure would help clarify the mechanistic basis of the observed behavior.

### 4.5 Limitations and Future Perspectives

Some limitations of our study should be acknowledged, along with directions for future research. First, we did not observe reduced hedonic responding or decreased social exploration in SERT^−/−^ females, behaviors that index motivational deficits and anhedonia, core features of human depression. The absence of these effects suggests that not all depressive‐like dimensions were captured in this SERT‐deficient model. Future studies employing higher sucrose concentrations may clarify whether the lack of genotype effects reflects the limited sensitivity of the low concentrations used here. In addition, assessing social anhedonia could provide a more comprehensive characterization of motivational alterations in SERT^−/−^ rodents. Second, the assessment of maternal care was constrained by the limited visibility of the nest and fixed observation timeframes. As a result, we could not examine caregiving behaviors before or immediately after pup separation, which may be critical periods for maternal anticipation and reunion‐related interactions. More fine‐grained temporal analyses of maternal behavior could help determine how caregiving dynamics modulate offspring behavioral outcomes and contribute to MS‐related effects. Additionally, the exact timing of dam body weight measurements relative to the daily maternal separation procedure was not documented. Consequently, we cannot determine whether the apparent convergence in body weight at PND16 reflects recovery following the final maternal separation session or differences in the timing of the measurements relative to the separation procedure. Future studies should systematically record the timing of body weight assessments in relation to the maternal separation protocol to facilitate interpretation of these temporal changes. Third, the choice of a 15‐min separation protocol for the control group, essential to control for the stress of procedural handling, may have shifted these animals from a completely undisturbed baseline via early handling effects, potentially reducing the contrast between the control and MS groups. Fourth, we attempted to minimize cumulative testing effects by placing the most stressful procedure (FST) at the end of the battery and including a one‐week washout period between consecutive tests. The ordering of the remaining tasks was chosen to prioritize the assessment of baseline anxiety and novelty responses before repeated handling and testing‐room habituation could attenuate these behaviors. However, our testing sequence, which prioritized assessing baseline emotional reactivity with the least possible interference from repeated handling and experimental room habituation, may have influenced performance on the later cognitive and social tests. Future studies employing separate cohorts for distinct behavioral domains would be valuable to cross‐validate these findings without the confounding variable of a sequential test battery. Lastly, although SERT^+/−^ rodents share mechanistic similarities with human 5‐HTTLPR short‐allele carriers [23], and are therefore valuable for studying GXE interactions, SERT^+/−^ females did not show increased vulnerability to depression‐related behaviors across MS conditions. This may indicate that postnatal ELS, as modeled by MS in the present study, alone is not sufficient to capture the severity of human early‐life adversity in females with reduced SERT expression. Future studies could explore whether ELS before birth, or the accumulation of maternal stress prior to conception, may capture the intergenerational transmission of vulnerability and produce more persistent depressive‐like phenotypes in the offspring.

## 5 Conclusion

Neither predictable nor unpredictable MS exposure increased depressive‐ and anxiety‐like behaviors in SERT‐deficient female rats. Instead, variations in SERT expression modulated the behavioral consequences of ELS, depending on both the specific genotype and the behavioral domain evaluated. Overall, unpredictable MS attenuated anxiety‐like behavior in SERT^−/−^ females, while unpredictable 3 h/day of MS (MSU180) differentially affected cognitive performance depending on SERT genotype, and prolonged predictable MS (MS360) reduced passive stress coping in SERT^+/−^ females. Our findings indicate that reduced or absent SERT expression does not consistently predispose female rats to depression‐related behavioral phenotypes. Rather, the behavioral effects of reduced SERT expression varied across behavioral domains and depended on the type of early‐life maternal separation experienced. This highlights the importance of conceptualizing SERT genotype as a moderator, rather than a determinant, of stress vulnerability. Additionally, MS duration and predictability distinctly influence behavioral outcomes, indicating that ELS paradigms are not interchangeable, and that specific features of adversity critically shape long‐term behavioral trajectories. The SERT × ELS interaction effects observed here further suggest that females may exhibit a degree of resilience to developing depression‐related behaviors, despite their increased biological sensitivity to stress. These findings raise important questions about sex‐specific mechanisms of stress adaptation and underscore the need to refine preclinical models of early life adversity. In particular, they point to the necessity of considering sex as a biological variable when modeling anxiety‐ and depression‐like phenotypes, to better capture the sex‐specific variability in symptom expression and comorbidity observed in human mood disorders.

## Funding

This work was supported by the Colombian Ministry of Science, Technology and Innovation (MINCIENCIAS, scholarship program No. 860) but it had no role in study design, data collection, data analysis, data interpretation, or the writing of the report.

## Conflicts of Interest

The authors declare no conflicts of interest.

## Supporting information


**Figure S1:** Frequency (A) and time (B) in the center of the OFT of control and maternally separated SERT^+/+^, SERT^+/−^ and SERT^−/−^ females (mean ± SEM). CTR: control handled animals; MS360: 6 h/day of predictable MS; MS180: 3 h/day of predictable MS; MSU180: 3 h/day of unpredictable MS; MSUS180: 3 h/day of unpredictable MS combined with maternal stress. Final sample sizes per group: SERT^+/+^ (CTR = 10; MS360 = 9; MS180 = 9; MSU180 = 7; MSUS180 = 7); SERT^+/−^ (CTR = 9; MS360 = 9; MS180 = 9; MSU180 = 9; MSUS180 = 9); SERT^−/−^ (CTR = 7; MS360 = 8; MS180 = 9; MSU180 = 9; MSUS180 = 8).
**Table S1:** Litter representation across experimental groups.
**Table S2:** Sample sizes (*n*), means, and SEMs of offspring body weight (grams) across experimental groups by postnatal day.
**Table S3:** Final sample sizes (*n*), means, and SEMs for offspring sucrose preference (%) and sucrose intake (mg/g rat) across experimental groups.

## Data Availability

The data that support the findings of this study are available from the corresponding author upon reasonable request.

## References

[1] S. L. James , D. Abate , K. H. Abate , et al., “Global, Regional, and National Incidence, Prevalence, and Years Lived With Disability for 354 Diseases and Injuries for 195 Countries and Territories, 1990–2017: A Systematic Analysis for the Global Burden of Disease Study 2017,” Lancet 392, no. 10159 (2018): 1789–1858, 10.1016/S0140-6736(18)32279-7. PMC6227754 30496104

[2] W. G. ter Meulen , S. Draisma , A. M. van Hemert , et al., “Depressive and Anxiety Disorders in Concert‐A Synthesis of Findings on Comorbidity in the NESDA Study,” Journal of Affective Disorders 284 (2021): 85–97, 10.1016/j.jad.2021.02.004. 33588240

[3] GBD 2019 Mental Disorders Collaborators , “Global, Regional, and National Burden of 12 Mental Disorders in 204 Countries and Territories, 1990–2019: A Systematic Analysis for the Global Burden of Disease Study 2019,” Lancet 399, no. 10334 (2022): 1510–1529, 10.1016/S0140-6736(21)02143-7. PMC8776563 35026139

[4] A. M. Buch and C. Liston , “Dissecting Diagnostic Heterogeneity in Depression by Integrating Neuroimaging and Genetics,” Neuropsychopharmacology 46, no. 1 (2021): 156–175, 10.1038/s41386-020-00789-3. PMC7688954 32781460

[5] D. W. Belsky and B. W. Domingue , “Progress and Challenges in GxE Research on Depression,” American Journal of Psychiatry 180, no. 2 (2023): 111–113, 10.1176/appi.ajp.20221039. 36722121

[6] Y.‐K. Kim , B.‐J. Ham , and K.‐M. Han , “Interactive Effects of Genetic Polymorphisms and Childhood Adversity on Brain Morphologic Changes in Depression,” Progress in Neuro‐Psychopharmacology and Biological Psychiatry 91 (2019): 4–13, 10.1016/j.pnpbp.2018.03.009. 29535036

[7] M. Kouris and R. Kumsta , “Polygenic Scores in the Study of Gene‐Environment Interplay and Stress‐Related Psychopathology,” Frontiers in Psychiatry 17 (2026): 1736828, 10.3389/fpsyt.2026.1736828. PMC13000120 41868848

[8] A. Caspi , K. Sugden , T. E. Moffitt , et al., “Influence of Life Stress on Depression: Moderation by a Polymorphism in the 5‐HTT Gene,” Science 301, no. 5631 (2003): 386–389, 10.1126/science.1083968. 12869766

[9] K.‐P. Lesch , D. Bengel , A. Heils , et al., “Association of Anxiety‐Related Traits With a Polymorphism in the Serotonin Transporter Gene Regulatory Region,” Science 274, no. 5292 (1996): 1527–1531, 10.1126/science.274.5292.1527. 8929413

[10] D. Bleys , P. Luyten , B. Soenens , and S. Claes , “Gene‐Environment Interactions Between Stress and 5‐HTTLPR in Depression: A Meta‐Analytic Update,” Journal of Affective Disorders 226 (2018): 339–345, 10.1016/j.jad.2017.09.050. 29031184

[11] K. Karg , M. Burmeister , K. Shedden , and S. Sen , “The Serotonin Transporter Promoter Variant (5‐HTTLPR), Stress, and Depression Meta‐Analysis Revisited: Evidence of Genetic Moderation,” Archives of General Psychiatry 68, no. 5 (2011): 444–454, 10.1001/archgenpsychiatry.2010.189. PMC3740203 21199959

[12] C. F. Sharpley , S. K. A. Palanisamy , N. S. Glyde , P. W. Dillingham , and L. L. Agnew , “An Update on the Interaction Between the Serotonin Transporter Promoter Variant (5‐HTTLPR), Stress and Depression, Plus an Exploration of Non‐Confirming Findings,” Behavioural Brain Research 273 (2014): 89–105, 10.1016/j.bbr.2014.07.030. 25078292

[13] M. R. Munafò , C. Durrant , G. Lewis , and J. Flint , “Gene X Environment Interactions at the Serotonin Transporter Locus,” Biological Psychiatry 65, no. 3 (2009): 211–219, 10.1016/j.biopsych.2008.06.009. 18691701

[14] N. Risch , R. Herrell , T. Lehner , et al., “Interaction Between the Serotonin Transporter Gene (5‐HTTLPR), Stressful Life Events, and Risk of Depression: A Meta‐Analysis,” JAMA 301, no. 23 (2009): 2462–2471, 10.1001/jama.2009.878. PMC2938776 19531786

[15] R. C. Culverhouse , N. L. Saccone , A. C. Horton , et al., “Collaborative Meta‐Analysis Finds no Evidence of a Strong Interaction Between Stress and 5‐HTTLPR Genotype Contributing to the Development of Depression,” Molecular Psychiatry 23, no. 1 (2018): 133–142, 10.1038/mp.2017.44. PMC5628077 28373689

[16] R. Border , E. C. Johnson , L. M. Evans , et al., “No Support for Historical Candidate Gene or Candidate Gene‐By‐Interaction Hypotheses for Major Depression Across Multiple Large Samples,” American Journal of Psychiatry 176, no. 5 (2019): 376–387, 10.1176/appi.ajp.2018.18070881. PMC6548317 30845820

[17] J. L. Jenness , B. L. Hankin , J. R. Z. Abela , J. F. Young , and A. Smolen , “Chronic Family Stress Interacts With 5‐HTTLPR to Predict Prospective Depressive Symptoms Among Youth,” Depression and Anxiety 28, no. 12 (2011): 1074–1080, 10.1002/da.20904. PMC3229659 22058064

[18] J. N. Vrijsen , I. Tendolkar , A. Arias‐Vásquez , et al., “Interaction of the 5‐HTTLPR and Childhood Trauma Influences Memory Bias in Healthy Individuals,” Journal of Affective Disorders 186 (2015): 83–89, 10.1016/j.jad.2015.06.008. 26232751

[19] G. Özçürümez , H. T. Yurdakul , Y. Terzi , N. Direk , A. Essizoglu , and F. Sahin , “The Interaction Between Childhood Maltreatment and Serotonin Transporter Gene in Recurrent Major Depressive Disorder: A Clinical Sample,” Archives of Neuropsychiatry 56 (2018): 110–114, 10.29399/npa.23325. PMC6563855 31223242

[20] D. Bengel , D. L. Murphy , A. M. Andrews , et al., “Altered Brain Serotonin Homeostasis and Locomotor Insensitivity to 3,4‐Methylenedioxymethamphetamine (“Ecstasy”) in Serotonin Transporter‐Deficient Mice,” Molecular Pharmacology 53, no. 4 (1998): 649–655, 10.1124/mol.53.4.649. 9547354

[21] B. M. Smits , J. B. Mudde , J. van de Belt , et al., “Generation of Gene Knockouts and Mutant Models in the Laboratory Rat by ENU‐Driven Target‐Selected Mutagenesis,” Pharmacogenetics and Genomics 16, no. 3 (2006): 159–169, 10.1097/01.fpc.0000184960.82903.8f. 16495775

[22] A. V. Kalueff , J. D. A. Olivier , L. J. P. Nonkes , and J. R. Homberg , “Conserved Role for the Serotonin Transporter Gene in Rat and Mouse Neurobehavioral Endophenotypes,” Neuroscience & Biobehavioral Reviews 34, no. 3 (2010): 373–386, 10.1016/j.neubiorev.2009.08.003. 19698744

[23] J. R. Homberg , J. D. A. Olivier , B. M. G. Smits , et al., “Characterization of the Serotonin Transporter Knockout Rat: A Selective Change in the Functioning of the Serotonergic System,” Neuroscience 146, no. 4 (2007): 1662–1676, 10.1016/j.neuroscience.2007.03.030. 17467186

[24] D. J. Houwing , B. Buwalda , E. A. van der Zee , S. F. de Boer , and J. D. A. Olivier , “The Serotonin Transporter and Early Life Stress: Translational Perspectives,” Frontiers in Cellular Neuroscience 11 (2017): 117, 10.3389/fncel.2017.00117. PMC5405142 28491024

[25] D. Frank , A. Zlotnik , O. Kofman , et al., “Early Life Stress Induces Submissive Behavior in Adult Rats,” Behavioural Brain Research 372 (2019): 112025, 10.1016/j.bbr.2019.112025. 31254880

[26] M. P. Leussis , N. Freund , H. C. Brenhouse , B. S. Thompson , and S. L. Andersen , “Depressive‐Like Behavior in Adolescents After Maternal Separation: Sex Differences, Controllability, and GABA,” Developmental Neuroscience 34, no. 2–3 (2012): 210–217, 10.1159/000339162. PMC5267293 22776911

[27] L. Lundgaard Donovan , K. Henningsen , A. Flou Kristensen , O. Wiborg , J. D. Nieland , and J. Lichota , “Maternal Separation Followed by Chronic Mild Stress in Adulthood Is Associated With Concerted Epigenetic Regulation of AP‐1 Complex Genes,” Journal of Personalized Medicine 11, no. 3 (2021): 209, 10.3390/jpm11030209. PMC8002051 33809485

[28] T. B. Franklin , H. Russig , I. C. Weiss , et al., “Epigenetic Transmission of the Impact of Early Stress Across Generations,” Biological Psychiatry 68, no. 5 (2010): 408–415, 10.1016/j.biopsych.2010.05.036. 20673872

[29] D. J. Houwing , A. S. Ramsteijn , I. W. Riemersma , and J. D. A. Olivier , “Maternal Separation Induces Anhedonia in Female Heterozygous Serotonin Transporter Knockout Rats,” Behavioural Brain Research 356 (2019): 204–207, 10.1016/j.bbr.2018.08.031. 30176268

[30] R. H. A. van der Doelen , T. Kozicz , and J. R. Homberg , “Adaptive Fitness; Early Life Adversity Improves Adult Stress Coping in Heterozygous Serotonin Transporter Knockout Rats,” Molecular Psychiatry 18, no. 12 (2013): 1244–1245, 10.1038/mp.2012.186. 23319004

[31] R. A. Millstein and A. Holmes , “Effects of Repeated Maternal Separation on Anxiety‐ and Depression‐Related Phenotypes in Different Mouse Strains,” Neuroscience & Biobehavioral Reviews 31, no. 1 (2007): 3–17, 10.1016/j.neubiorev.2006.05.003. 16950513

[32] S. G. Tractenberg , M. L. Levandowski , L. A. de Azeredo , et al., “An Overview of Maternal Separation Effects on Behavioural Outcomes in Mice: Evidence From a Four‐Stage Methodological Systematic Review,” Neuroscience & Biobehavioral Reviews 68 (2016): 489–503, 10.1016/j.neubiorev.2016.06.021. 27328784

[33] R. L. Alves , C. C. Portugal , T. Summavielle , F. Barbosa , and A. Magalhães , “Maternal Separation Effects on Mother Rodents' Behaviour: A Systematic Review,” Neuroscience & Biobehavioral Reviews 117 (2020): 98–109, 10.1016/j.neubiorev.2019.09.008. 31499082

[34] O. V. Burenkova and E. L. Grigorenko , “The Role of Epigenetic Mechanisms in the Long‐Term Effects of Early‐Life Adversity and Mother‐Infant Relationship on Physiology and Behavior of Offspring in Laboratory Rats and Mice,” Developmental Psychobiology 66, no. 3 (2024): e22479, 10.1002/dev.22479. PMC10959231 38470450

[35] P. R. Albert , “Why Is Depression More Prevalent in Women?,” Journal of Psychiatry and Neuroscience 40, no. 4 (2015): 219–221, 10.1503/jpn.150205. PMC4478054 26107348

[36] R. H. Salk , J. S. Hyde , and L. Y. Abramson , “Gender Differences in Depression in Representative National Samples: Meta‐Analyses of Diagnoses and Symptoms,” Psychological Bulletin 143, no. 8 (2017): 783–822, 10.1037/bul0000102. PMC5532074 28447828

[37] J. M. Platt , L. Bates , J. Jager , K. A. McLaughlin , and K. M. Keyes , “Is the US Gender Gap in Depression Changing Over Time? A Meta‐Regression,” American Journal of Epidemiology 190, no. 7 (2021): 1190–1206, 10.1093/aje/kwab002. PMC8484777 33423055

[38] World Health Organization , Depression and Other Common Mental Disorders: Global Health Estimates (World Health Organization, 2017).

[39] J. Corcoran , I. Marinescu , C. Vogelsang , and J. C. Kim , “Prevalence of Depression During Pregnancy and Postpartum Periods in Low‐Income Women in Developed Countries,” Journal of Public Health 30, no. 10 (2022): 2473–2482, 10.1007/s10389-021-01662-3.

[40] G. R. Rinne , E. P. Davis , N. E. Mahrer , et al., “Maternal Depressive Symptom Trajectories From Preconception Through Postpartum: Associations With Offspring Developmental Outcomes in Early Childhood,” Journal of Affective Disorders 309 (2022): 105–114, 10.1016/j.jad.2022.04.116. PMC10024939 35461817

[41] T. L. Bale and C. N. Epperson , “Sex as a Biological Variable: Who, What, When, Why, and How,” Neuropsychopharmacology 42, no. 2 (2017): 386–396, 10.1038/npp.2016.215. PMC5399243 27658485

[42] R. S. Eid , A. R. Gobinath , and L. A. M. Galea , “Sex Differences in Depression: Insights From Clinical and Preclinical Studies,” Progress in Neurobiology 176 (2019): 86–102, 10.1016/j.pneurobio.2019.01.006. 30721749

[43] S. El Aidy , A. S. Ramsteijn , F. Dini‐Andreote , et al., “Serotonin Transporter Genotype Modulates the Gut Microbiota Composition in Young Rats, an Effect Augmented by Early Life Stress,” Frontiers in Cellular Neuroscience 11 (2017): 222, 10.3389/fncel.2017.00222. PMC5540888 28824378

[44] J. P. ter Horst , E. R. de Kloet , H. Schächinger , and M. S. Oitzl , “Relevance of Stress and Female Sex Hormones for Emotion and Cognition,” Cellular and Molecular Neurobiology 32, no. 5 (2012): 725–735, 10.1007/s10571-011-9774-2. PMC3377901 22113371

[45] Ö. S. Çevik , D. D. Yıldırım , C. Uzun , and E. Horata , “Contribution of Distinctive Outcome Measures to the Assessment of Anxiety in the Open Field: A Meta‐Analysis of Factors Mediating Open‐Field Test Variability in Rodent Models of Anxiety,” Behavioural Brain Research 490 (2025): 115612, 10.1016/j.bbr.2025.115612. 40311939

[46] R. C. S. R. Danduga and P. K. Kola , “Elevated Plus Maze for Assessment of Anxiety and Memory in Rodents,” Methods in Molecular Biology (Clifton, N.J.) 2761 (2024): 93–96, 10.1007/978-1-0716-3662-6_8. 38427232

[47] S. S. Moy , J. J. Nadler , A. Perez , et al., “Sociability and Preference for Social Novelty in Five Inbred Strains: An Approach to Assess Autistic‐Like Behavior in Mice,” Genes, Brain, and Behavior 3, no. 5 (2004): 287–302, 10.1111/j.1601-1848.2004.00076.x. 15344922

[48] M. J. Primo , D. Fonseca‐Rodrigues , A. Almeida , P. M. Teixeira , and F. Pinto‐Ribeiro , “Sucrose Preference Test: A Systematic Review of Protocols for the Assessment of Anhedonia in Rodents,” European Neuropsychopharmacology 77 (2023): 80–92, 10.1016/j.euroneuro.2023.08.496. 37741164

[49] L. F. Hiew , S. Khairuddin , L. Aquili , et al., “Behavioural Responses of Anxiety in Aversive and Non‐Aversive Conditions Between Young and Aged Sprague‐Dawley Rats,” Behavioural Brain Research 385 (2020): 112559, 10.1016/j.bbr.2020.112559. 32097707

[50] R. E. Clark , S. M. Zola , and L. R. Squire , “Impaired Recognition Memory in Rats After Damage to the Hippocampus,” Journal of Neuroscience 20, no. 23 (2000): 8853–8860, 10.1523/JNEUROSCI.20-23-08853.2000. PMC6773055 11102494

[51] M. L. Molendijk and E. R. de Kloet , “Immobility in the Forced Swim Test Is Adaptive and Does Not Reflect Depression,” Psychoneuroendocrinology 62 (2015): 389–391, 10.1016/j.psyneuen.2015.08.028. 26386543

[52] R Core Team , R: A Language and Environment for Statistical Computing (R Foundation for Statistical Computing, 2024), 10.32614/r.manuals.

[53] S. Akkerman , J. Prickaerts , H. W. M. Steinbusch , and A. Blokland , “Object Recognition Testing: Statistical Considerations,” Behavioural Brain Research 232, no. 2 (2012): 317–322, 10.1016/j.bbr.2012.03.024. 22481080

[54] K. L. Tamashiro , R. R. Sakai , C. A. Shively , I. N. Karatsoreos , and L. P. Reagan , “Chronic Stress, Metabolism, and Metabolic Syndrome,” Stress 14, no. 5 (2011): 468–474, 10.3109/10253890.2011.606341. 21848434

[55] C. C. Josefson , L. De Moura Pereira , and A. L. Skibiel , “Chronic Stress Decreases Lactation Performance,” Integrative and Comparative Biology 63, no. 3 (2023): 557–568, 10.1093/icb/icad044. 37253624

[56] J. Hoch , N. Burkhard , S. Zhang , et al., “Serotonin Transporter‐Deficient Mice Display Enhanced Adipose Tissue Inflammation After Chronic High‐Fat Diet Feeding,” Frontiers in Immunology 14 (2023): 1184010, 10.3389/fimmu.2023.1184010. PMC10372416 37520561

[57] A. Holmes , Q. Li , D. L. Murphy , E. Gold , and J. N. Crawley , “Abnormal Anxiety‐Related Behavior in Serotonin Transporter Null Mutant Mice: The Influence of Genetic Background: Anxiety‐Like Behavior in 5‐HTT Null Mutant Mice,” Genes, Brain, and Behavior 2, no. 6 (2003): 365–380, 10.1046/j.1601-1848.2003.00050.x. 14653308

[58] M. Tanaka , A. Sato , S. Kasai , et al., “Brain Hyperserotonemia Causes Autism‐Relevant Social Deficits in Mice,” Molecular Autism 9, no. 1 (2018): 60, 10.1186/s13229-018-0243-3. PMC6258166 30498565

[59] N. C. Coimbra , T. Paschoalin‐Maurin , G. S. Bassi , et al., “Critical Neuropsychobiological Analysis of Panic Attack‐ and Anticipatory Anxiety‐Like Behaviors in Rodents Confronted With Snakes in Polygonal Arenas and Complex Labyrinths: A Comparison to the Elevated Plus‐ and T‐Maze Behavioral Tests,” Revista Brasileira de Psiquiatria 39, no. 1 (2017): 72–83, 10.1590/1516-4446-2015-1895. PMC7112733 28177062

[60] D. J. Houwing , L. Staal , J. M. Swart , et al., “Subjecting Dams to Early Life Stress and Perinatal Fluoxetine Treatment Differentially Alters Social Behavior in Young and Adult Rat Offspring,” Frontiers in Neuroscience 13 (2019): 229, 10.3389/fnins.2019.00229. PMC6423179 30914920

[61] J. Golebiowska , M. Hołuj , A. Potasiewicz , et al., “Serotonin Transporter Deficiency Alters Socioemotional Ultrasonic Communication in Rats,” Scientific Reports 9, no. 1 (2019): 20283, 10.1038/s41598-019-56629-y. PMC6937290 31889084

[62] A. Holmes , “Evaluation of Antidepressant‐Related Behavioral Responses in Mice Lacking the Serotonin Transporter,” Neuropsychopharmacology 27, no. 6 (2002): 914–923, 10.1016/S0893-133X(02)00374-3. 12464448

[63] J. D. A. Olivier , M. G. C. Van Der Hart , R. P. L. Van Swelm , et al., “A Study in Male and Female 5‐HT Transporter Knockout Rats: An Animal Model for Anxiety and Depression Disorders,” Neuroscience 152, no. 3 (2008): 573–584, 10.1016/j.neuroscience.2007.12.032. 18295409

[64] H. Shoji , K. Ikeda , and T. Miyakawa , “Behavioral Phenotype, Intestinal Microbiome, and Brain Neuronal Activity of Male Serotonin Transporter Knockout Mice,” Molecular Brain 16, no. 1 (2023): 32, 10.1186/s13041-023-01020-2. PMC10061809 36991468

[65] J. M. Koolhaas , S. M. Korte , S. F. De Boer , et al., “Coping Styles in Animals: Current Status in Behavior and Stress‐Physiology,” Neuroscience & Biobehavioral Reviews 23, no. 7 (1999): 925–935, 10.1016/S0149-7634(99)00026-3. 10580307

[66] J. F. Cryan and A. Holmes , “The Ascent of Mouse: Advances in Modelling Human Depression and Anxiety,” Nature Reviews Drug Discovery 4, no. 9 (2005): 775–790, 10.1038/nrd1825. 16138108

[67] J. M. Weiss and C. D. Kilts , “Animal Models of Depression and Schizophrenia,” in Textbook of Psychopharmacology, ed. M. A. Schatzberg and C. B. Nemeroff (American Psychiatric Press, 1998), 89–131.

[68] K. Schraut , N. Leibold , M. T. Weidner , et al., “P.1.H.040 Differential Effects of Prenatal Stress in Female 5‐HTT Deficient Mice: Towards Molecular Mechanisms of Resilience,” European Neuropsychopharmacology 24 (2014): S292–S293, 10.1016/S0924-977X(14)70463-8. 25195605

[69] A. V. Kalueff , P. S. Gallagher , and D. L. Murphy , “Are Serotonin Transporter Knockout Mice ‘Depressed’?: Hypoactivity but no Anhedonia,” Neuroreport 17, no. 12 (2006): 1347–1351, 10.1097/01.wnr.0000230514.08962.76. 16951583

[70] M. T. Perona , S. Waters , F. S. Hall , et al., “Animal Models of Depression in Dopamine, Serotonin, and Norepinephrine Transporter Knockout Mice: Prominent Effects of Dopamine Transporter Deletions,” Behavioural Pharmacology 19, no. 5–6 (2008): 566–574, 10.1097/fbp.0b013e32830cd80f. PMC2644662 18690111

[71] G. Sbrini , P. Brivio , K. Bosch , J. R. Homberg , and F. Calabrese , “Enrichment Environment Positively Influences Depression‐ and Anxiety‐Like Behavior in Serotonin Transporter Knockout Rats Through the Modulation of Neuroplasticity, Spine, and GABAergic Markers,” Genes 11, no. 11 (2020): 1248, 10.3390/genes11111248. PMC7690660 33114023

[72] D. M. Diniz , F. Calabrese , P. Brivio , M. A. Riva , J. Grandjean , and J. R. Homberg , “BDNF Overexpression in the Ventral Hippocampus Promotes Antidepressant‐ and Anxiolytic‐Like Activity in Serotonin Transporter Knockout Rats,” International Journal of Molecular Sciences 22, no. 9 (2021): 5040, 10.3390/ijms22095040. PMC8126235 34068707

[73] Q. Li , C. Wichems , A. Heils , L. D. Van De Kar , K. P. Lesch , and D. L. Murphy , “Reduction of 5‐Hydroxytryptamine (5‐HT)(1A)‐Mediated Temperature and Neuroendocrine Responses and 5‐HT(1A) Binding Sites in 5‐HT Transporter Knockout Mice,” Journal of Pharmacology and Experimental Therapeutics 291, no. 3 (1999): 999–1007. 10565817

[74] Q. Li , C. H. Wichems , L. Ma , L. D. Van de Kar , F. Garcia , and D. L. Murphy , “Brain Region‐Specific Alterations of 5‐HT2A and 5‐HT2C Receptors in Serotonin Transporter Knockout Mice,” Journal of Neurochemistry 84, no. 6 (2003): 1256–1265, 10.1046/j.1471-4159.2003.01607.x. 12614326

[75] C. Mannoury la Cour , C. Boni , N. Hanoun , K. P. Lesch , M. Hamon , and L. Lanfumey , “Functional Consequences of 5‐HT Transporter Gene Disruption on 5‐HT(1a) Receptor‐Mediated Regulation of Dorsal Raphe and Hippocampal Cell Activity,” Journal of Neuroscience 21, no. 6 (2001): 2178–2185, 10.1523/jneurosci.21-06-02178.2001. PMC6762595 11245702

[76] G. Guidotti , F. Calabrese , F. Auletta , et al., “Developmental Influence of the Serotonin Transporter on the Expression of npas4 and GABAergic Markers: Modulation by Antidepressant Treatment,” Neuropsychopharmacology 37, no. 3 (2012): 746–758, 10.1038/npp.2011.252. PMC3260971 22012473

[77] R. Molteni , A. Cattaneo , F. Calabrese , et al., “Reduced Function of the Serotonin Transporter Is Associated With Decreased Expression of BDNF in Rodents as Well as in Humans,” Neurobiology of Disease 37, no. 3 (2010): 747–755, 10.1016/j.nbd.2009.12.014. 20034565

[78] P. C. Bendis , S. Zimmerman , A. Onisiforou , P. Zanos , and P. Georgiou , “The Impact of Estradiol on Serotonin, Glutamate, and Dopamine Systems,” Frontiers in Neuroscience 18 (2024): 1348551, 10.3389/fnins.2024.1348551. PMC10998471 38586193

[79] M. Le Saux and T. Di Paolo , “Changes in 5‐HT1A Receptor Binding and G‐Protein Activation in the Rat Brain After Estrogen Treatment: Comparison With Tamoxifen and Raloxifene,” Journal of Psychiatry & Neuroscience: JPN 30, no. 2 (2005): 110–117. PMC551165 15798786

[80] M. K. Osterlund and Y. L. Hurd , “Acute 17 Beta‐Estradiol Treatment Down‐Regulates Serotonin 5HT1A Receptor mRNA Expression in the Limbic System of Female Rats,” Brain Research. Molecular Brain Research 55, no. 1 (1998): 169–172, 10.1016/s0169-328x(98)00018-7. 9645972

[81] E. Choleris , J. A. Gustafsson , K. S. Korach , L. J. Muglia , D. W. Pfaff , and S. Ogawa , “An Estrogen‐Dependent Four‐Gene Micronet Regulating Social Recognition: A Study With Oxytocin and Estrogen Receptor‐Alpha and ‐Beta Knockout Mice,” PNAS 100, no. 10 (2003): 6192–6197, 10.1073/pnas.0631699100. PMC156348 12730370

[82] A. A. Krentzel , J. A. Willett , A. G. Johnson , and J. Meitzen , “Estrogen Receptor Alpha, G‐Protein Coupled Estrogen Receptor 1, and Aromatase: Developmental, Sex, and Region‐Specific Differences Across the Rat Caudate‐Putamen, Nucleus Accumbens Core and Shell,” Journal of Comparative Neurology 529, no. 4 (2021): 786–801, 10.1002/cne.24978. PMC7775873 32632943

[83] D. C. Blanchard , G. Griebel , and R. J. Blanchard , “Mouse Defensive Behaviors: Pharmacological and Behavioral Assays for Anxiety and Panic,” Neuroscience and Biobehavioral Reviews 25, no. 3 (2001): 205–218, 10.1016/s0149-7634(01)00009-4. 11378177

[84] L. R. Berardo , M. C. Fabio , and R. M. Pautassi , “Post‐Weaning Environmental Enrichment, but Not Chronic Maternal Isolation, Enhanced Ethanol Intake During Periadolescence and Early Adulthood,” Frontiers in Behavioral Neuroscience 10 (2016): 195, 10.3389/fnbeh.2016.00195. PMC5061821 27790100

[85] M. Kalinichev , K. W. Easterling , and S. G. Holtzman , “Early Neonatal Experience of Long‐Evans Rats Results in Long‐Lasting Changes in Reactivity to a Novel Environment and Morphine‐Induced Sensitization and Tolerance,” Neuropsychopharmacology 27, no. 4 (2002): 518–533, 10.1016/S0893-133X(02)00326-3. 12377389

[86] J. H. van Heerden , V. Russell , A. Korff , D. J. Stein , and N. Illing , “Evaluating the Behavioural Consequences of Early Maternal Separation in Adult C57BL/6 Mice; the Importance of Time,” Behavioural Brain Research 207, no. 2 (2010): 332–342, 10.1016/j.bbr.2009.10.015. 19850084

[87] O. Stupart , T. W. Robbins , and J. W. Dalley , ““The Wrong Tools for the Right Job”: A Critical Meta‐Analysis of Traditional Tests to Assess Behavioural Impacts of Maternal Separation,” Psychopharmacology 240, no. 11 (2023): 2239–2256, 10.1007/s00213-022-06275-6. PMC10593619 36418564

[88] R. L. Alves , C. C. Portugal , I. M. Lopes , et al., “Maternal Stress and Vulnerability to Depression: Coping and Maternal Care Strategies and Its Consequences on Adolescent Offspring,” Translational Psychiatry 12, no. 1 (2022): 463, 10.1038/s41398-022-02220-5. PMC9636172 36333302

[89] P. Pan , A. S. Fleming , D. Lawson , J. M. Jenkins , and P. O. McGowan , “Within‐ and Between‐Litter Maternal Care Alter Behavior and Gene Regulation in Female Offspring,” Behavioral Neuroscience 128, no. 6 (2014): 736–748, 10.1037/bne0000014. 25244085

[90] S. Toki , S. Morinobu , A. Imanaka , S. Yamamoto , S. Yamawaki , and K. Honma , “Importance of Early Lighting Conditions in Maternal Care by Dam as Well as Anxiety and Memory Later in Life of Offspring,” European Journal of Neuroscience 25, no. 3 (2007): 815–829, 10.1111/j.1460-9568.2007.05288.x. 17328777

[91] Y. Horii , S. Nakajima , S. Akieda‐Asai , R. Ohta , and M. Kawaguchi . “Maternal Traits During Lactation Period Reduce the Anxiety‐Related Behavior in Male Offspring: Results From a Fostering Study in Hatano Rats,” Physiology & Behavior 229 (2021): 113209, 10.1016/j.physbeh.2020.113209. 33080275

[92] V. Carola , G. Frazzetto , T. Pascucci , et al., “Identifying Molecular Substrates in a Mouse Model of the Serotonin Transporter × Environment Risk Factor for Anxiety and Depression,” Biological Psychiatry 63, no. 9 (2008): 840–846, 10.1016/j.biopsych.2007.08.013. 17949690

[93] J. D. Olivier , L. A. Jans , A. Blokland , et al., “Serotonin Transporter Deficiency in Rats Contributes to Impaired Object Memory,” Genes, Brain, and Behavior 8, no. 8 (2009): 829–834, 10.1111/j.1601-183X.2009.00530.x. 19740092

[94] J. D. Olivier , L. A. Jans , G. A. Korte‐Bouws , et al., “Acute Tryptophan Depletion Dose Dependently Impairs Object Memory in Serotonin Transporter Knockout Rats,” Psychopharmacology 200, no. 2 (2008): 243–254, 10.1007/s00213-008-1201-0. 18542930

[95] D. L. van den Hove , S. B. Jakob , K. G. Schraut , et al., “Differential Effects of Prenatal Stress in 5‐Htt Deficient Mice: Towards Molecular Mechanisms of Gene × Environment Interactions,” PLoS One 6, no. 8 (2011): e22715, 10.1371/journal.pone.0022715. PMC3155516 21857948

[96] Y. Kroeze , B. Dirven , S. Janssen , et al., “Perinatal Reduction of Functional Serotonin Transporters Results in Developmental Delay,” Neuropharmacology 109 (2016): 96–111, 10.1016/j.neuropharm.2016.05.012. 27208789

[97] A. Ennaceur and J. Delacour , “A New One‐Trial Test for Neurobiological Studies of Memory in Rats. 1: Behavioral Data,” Behavioural Brain Research 31, no. 1 (1988): 47–59, 10.1016/0166-4328(88)90157-x. 3228475

[98] M. Rocha , D. Wang , V. Avila‐Quintero , M. H. Bloch , and A. Kaffman , “Deficits in Hippocampal‐Dependent Memory Across Different Rodent Models of Early Life Stress: Systematic Review and Meta‐Analysis,” Translational Psychiatry 11, no. 1 (2021): 231, 10.1038/s41398-021-01352-4. PMC8058062 33879774

[99] Z. Jiang , Z. Zhu , M. Zhao , et al., “H3K9me2 Regulation of BDNF Expression in the Hippocampus and Medial Prefrontal Cortex Is Involved in the Depressive‐Like Phenotype Induced by Maternal Separation in Male Rats,” Psychopharmacology 238, no. 10 (2021): 2801–2813, 10.1007/s00213-021-05896-7. 34328517

[100] G. Talani , F. Biggio , A. A. Gorule , et al., “Sex‐Dependent Changes of Hippocampal Synaptic Plasticity and Cognitive Performance in C57BL/6J Mice Exposed to Neonatal Repeated Maternal Separation,” Neuropharmacology 222 (2023): 109301, 10.1016/j.neuropharm.2022.109301. 36336069

