# Supplementary material for: Effects of Serotonin Transporter Genotype and (Un)Predictable Maternal Separation on Affective, Cognitive, and Social Behaviors in Female Rats
Source: Genes Brain Behav. 2026 Oct 12;25(5):e70069. doi: 10.1111/gbb.70069 (PMC13655770; doi:10.1111/gbb.70069)
Supplement: Supplementary file 1 [file GBB-25-e70069-s001.docx]

**Supplementary Material**

**Suppl. Fig. S1.** Frequency (A) and time (B) in the center of the OFT of control and maternally separated SERT^+/+^, SERT^+/-^ and SERT^-/-^ females (mean±SEM). CTR: control handled animals; MS360: 6hr/day of predictable MS; MS180: 3hr/day of predictable MS; MSU180: 3hr/day of unpredictable MS; MSUS180: 3hr/day of unpredictable MS combined with maternal stress. Final sample sizes per group: SERT^+/+^ (CTR=10; MS360=9; MS180=9; MSU180=7; MSUS180=7); SERT^+/-^ (CTR=9; MS360=9; MS180=9; MSU180=9; MSUS180=9); SERT^-/-^ (CTR=7; MS360=8; MS180=9; MSU180=9; MSUS180=8).

**Suppl. Table S1.** Litter representation across experimental groups.

| **MS_type** | **genotype** | **Animals (n)** | **Contributing litters (n)** | **Litters with 1 animal** | **Litters with 2 animals** | **Litters with >2 animals** | **Maximum animals from one litter** |
| --- | --- | --- | --- | --- | --- | --- | --- |
| CTR | SERT+/+ | 10 | 6 | 2 | 4 | 0 | 2 |
| CTR | SERT+/- | 9 | 4 | 0 | 3 | 1 | 3 |
| CTR | SERT-/- | 6 | 6 | 6 | 0 | 0 | 1 |
| MS360 | SERT+/+ | 9 | 7 | 5 | 2 | 0 | 2 |
| MS360 | SERT+/- | 9 | 5 | 1 | 4 | 0 | 2 |
| MS360 | SERT-/- | 8 | 6 | 4 | 2 | 0 | 2 |
| MS180 | SERT+/+ | 9 | 6 | 3 | 3 | 0 | 2 |
| MS180 | SERT+/- | 9 | 6 | 3 | 3 | 0 | 2 |
| MS180 | SERT-/- | 9 | 6 | 4 | 1 | 1 | 3 |
| MSU180 | SERT+/+ | 7 | 6 | 5 | 1 | 0 | 2 |
| MSU180 | SERT+/- | 9 | 7 | 5 | 2 | 0 | 2 |
| MSU180 | SERT-/- | 9 | 6 | 3 | 3 | 0 | 2 |
| MSUS180 | SERT+/+ | 7 | 5 | 3 | 2 | 0 | 2 |
| MSUS180 | SERT+/- | 9 | 4 | 1 | 2 | 1 | 4 |
| MSUS180 | SERT-/- | 9 | 6 | 3 | 3 | 0 | 2 |

**Suppl. Table S2.** Sample sizes (*n*), means, and SEMs of offspring body weight (grams) across experimental groups by postnatal day.

| **Postnatal_Day** | **MS_type** | **genotype** | **n** | **mean** | **sem** |
| --- | --- | --- | --- | --- | --- |
| P7 | CTR | SERT+/+ | 10 | 13.55 | 0.33 |
| P7 | CTR | SERT+/- | 9 | 14.44 | 0.38 |
| P7 | CTR | SERT-/- | 7 | 13.10 | 1.00 |
| P7 | MS360 | SERT+/+ | 8 | 14.03 | 0.52 |
| P7 | MS360 | SERT+/- | 9 | 15.60 | 0.32 |
| P7 | MS360 | SERT-/- | 8 | 14.56 | 0.59 |
| P7 | MS180 | SERT+/+ | 9 | 14.52 | 0.19 |
| P7 | MS180 | SERT+/- | 9 | 15.03 | 0.28 |
| P7 | MS180 | SERT-/- | 9 | 14.57 | 0.42 |
| P7 | MSU180 | SERT+/+ | 7 | 14.90 | 0.59 |
| P7 | MSU180 | SERT+/- | 9 | 14.37 | 0.48 |
| P7 | MSU180 | SERT-/- | 9 | 14.91 | 0.26 |
| P7 | MSUS180 | SERT+/+ | 8 | 13.32 | 0.41 |
| P7 | MSUS180 | SERT+/- | 9 | 13.86 | 0.32 |
| P7 | MSUS180 | SERT-/- | 8 | 13.97 | 0.77 |
| P14 | CTR | SERT+/+ | 10 | 26.93 | 1.04 |
| P14 | CTR | SERT+/- | 9 | 28.29 | 1.13 |
| P14 | CTR | SERT-/- | 7 | 26.36 | 1.48 |
| P14 | MS360 | SERT+/+ | 9 | 26.82 | 1.09 |
| P14 | MS360 | SERT+/- | 9 | 28.64 | 0.97 |
| P14 | MS360 | SERT-/- | 8 | 28.35 | 1.17 |
| P14 | MS180 | SERT+/+ | 9 | 27.56 | 0.63 |
| P14 | MS180 | SERT+/- | 9 | 29.19 | 1.05 |
| P14 | MS180 | SERT-/- | 9 | 27.22 | 0.83 |
| P14 | MSU180 | SERT+/+ | 6 | 25.10 | 0.75 |
| P14 | MSU180 | SERT+/- | 9 | 26.20 | 1.07 |
| P14 | MSU180 | SERT-/- | 9 | 27.51 | 0.92 |
| P14 | MSUS180 | SERT+/+ | 8 | 25.90 | 0.81 |
| P14 | MSUS180 | SERT+/- | 9 | 25.02 | 0.51 |
| P14 | MSUS180 | SERT-/- | 8 | 26.59 | 0.94 |
| P21 | CTR | SERT+/+ | 10 | 41.50 | 0.97 |
| P21 | CTR | SERT+/- | 9 | 43.83 | 1.57 |
| P21 | CTR | SERT-/- | 7 | 36.31 | 1.56 |
| P21 | MS360 | SERT+/+ | 9 | 41.68 | 1.30 |
| P21 | MS360 | SERT+/- | 9 | 45.80 | 0.90 |
| P21 | MS360 | SERT-/- | 8 | 39.24 | 1.51 |
| P21 | MS180 | SERT+/+ | 9 | 42.92 | 1.30 |
| P21 | MS180 | SERT+/- | 9 | 44.36 | 1.54 |
| P21 | MS180 | SERT-/- | 8 | 35.04 | 0.51 |
| P21 | MSU180 | SERT+/+ | 7 | 41.27 | 1.95 |
| P21 | MSU180 | SERT+/- | 8 | 40.89 | 1.62 |
| P21 | MSU180 | SERT-/- | 7 | 35.61 | 0.62 |
| P21 | MSUS180 | SERT+/+ | 8 | 39.59 | 0.79 |
| P21 | MSUS180 | SERT+/- | 9 | 40.12 | 1.02 |
| P21 | MSUS180 | SERT-/- | 8 | 36.02 | 1.07 |
| P28 | CTR | SERT+/+ | 10 | 66.63 | 1.44 |
| P28 | CTR | SERT+/- | 9 | 68.53 | 2.60 |
| P28 | CTR | SERT-/- | 6 | 59.42 | 1.01 |
| P28 | MS360 | SERT+/+ | 9 | 67.46 | 2.29 |
| P28 | MS360 | SERT+/- | 9 | 73.20 | 1.63 |
| P28 | MS360 | SERT-/- | 7 | 64.13 | 1.17 |
| P28 | MS180 | SERT+/+ | 9 | 68.73 | 1.23 |
| P28 | MS180 | SERT+/- | 7 | 71.97 | 0.86 |
| P28 | MS180 | SERT-/- | 9 | 62.00 | 1.79 |
| P28 | MSU180 | SERT+/+ | 7 | 66.71 | 2.33 |
| P28 | MSU180 | SERT+/- | 9 | 67.44 | 2.32 |
| P28 | MSU180 | SERT-/- | 9 | 63.77 | 2.86 |
| P28 | MSUS180 | SERT+/+ | 8 | 64.30 | 1.48 |
| P28 | MSUS180 | SERT+/- | 9 | 67.19 | 2.00 |
| P28 | MSUS180 | SERT-/- | 8 | 64.55 | 1.58 |
| P35 | CTR | SERT+/+ | 10 | 103.00 | 2.12 |
| P35 | CTR | SERT+/- | 9 | 106.10 | 3.84 |
| P35 | CTR | SERT-/- | 6 | 94.98 | 1.55 |
| P35 | MS360 | SERT+/+ | 9 | 105.18 | 3.43 |
| P35 | MS360 | SERT+/- | 9 | 112.86 | 2.64 |
| P35 | MS360 | SERT-/- | 8 | 96.06 | 4.13 |
| P35 | MS180 | SERT+/+ | 9 | 107.50 | 1.39 |
| P35 | MS180 | SERT+/- | 9 | 105.79 | 2.36 |
| P35 | MS180 | SERT-/- | 9 | 92.70 | 4.13 |
| P35 | MSU180 | SERT+/+ | 7 | 103.79 | 2.62 |
| P35 | MSU180 | SERT+/- | 9 | 107.47 | 3.40 |
| P35 | MSU180 | SERT-/- | 9 | 98.43 | 3.93 |
| P35 | MSUS180 | SERT+/+ | 8 | 101.51 | 2.45 |
| P35 | MSUS180 | SERT+/- | 9 | 107.32 | 2.62 |
| P35 | MSUS180 | SERT-/- | 8 | 100.59 | 1.71 |
| P42 | CTR | SERT+/+ | 10 | 133.44 | 3.20 |
| P42 | CTR | SERT+/- | 9 | 135.18 | 3.27 |
| P42 | CTR | SERT-/- | 7 | 126.39 | 3.85 |
| P42 | MS360 | SERT+/+ | 9 | 132.42 | 3.49 |
| P42 | MS360 | SERT+/- | 9 | 142.09 | 2.17 |
| P42 | MS360 | SERT-/- | 7 | 125.23 | 1.72 |
| P42 | MS180 | SERT+/+ | 9 | 139.48 | 2.75 |
| P42 | MS180 | SERT+/- | 8 | 133.64 | 2.64 |
| P42 | MS180 | SERT-/- | 9 | 123.33 | 2.52 |
| P42 | MSU180 | SERT+/+ | 7 | 133.07 | 2.90 |
| P42 | MSU180 | SERT+/- | 9 | 137.66 | 3.19 |
| P42 | MSU180 | SERT-/- | 9 | 121.69 | 3.77 |
| P42 | MSUS180 | SERT+/+ | 8 | 131.75 | 3.29 |
| P42 | MSUS180 | SERT+/- | 9 | 135.42 | 3.38 |
| P42 | MSUS180 | SERT-/- | 8 | 126.67 | 3.14 |
| P49 | CTR | SERT+/+ | 10 | 153.28 | 3.88 |
| P49 | CTR | SERT+/- | 9 | 151.58 | 3.25 |
| P49 | CTR | SERT-/- | 7 | 142.53 | 4.53 |
| P49 | MS360 | SERT+/+ | 9 | 156.57 | 3.49 |
| P49 | MS360 | SERT+/- | 8 | 164.61 | 1.35 |
| P49 | MS360 | SERT-/- | 8 | 137.91 | 6.07 |
| P49 | MS180 | SERT+/+ | 9 | 159.30 | 2.91 |
| P49 | MS180 | SERT+/- | 9 | 149.11 | 5.71 |
| P49 | MS180 | SERT-/- | 9 | 143.86 | 1.48 |
| P49 | MSU180 | SERT+/+ | 7 | 152.80 | 3.18 |
| P49 | MSU180 | SERT+/- | 9 | 158.46 | 4.03 |
| P49 | MSU180 | SERT-/- | 9 | 141.38 | 3.30 |
| P49 | MSUS180 | SERT+/+ | 8 | 150.24 | 3.48 |
| P49 | MSUS180 | SERT+/- | 9 | 153.67 | 5.52 |
| P49 | MSUS180 | SERT-/- | 8 | 144.99 | 3.44 |
| P56 | CTR | SERT+/+ | 10 | 169.16 | 3.91 |
| P56 | CTR | SERT+/- | 9 | 171.06 | 3.23 |
| P56 | CTR | SERT-/- | 7 | 161.50 | 4.04 |
| P56 | MS360 | SERT+/+ | 9 | 172.02 | 3.11 |
| P56 | MS360 | SERT+/- | 9 | 181.11 | 2.98 |
| P56 | MS360 | SERT-/- | 8 | 155.85 | 6.22 |
| P56 | MS180 | SERT+/+ | 9 | 178.29 | 3.08 |
| P56 | MS180 | SERT+/- | 8 | 170.82 | 2.83 |
| P56 | MS180 | SERT-/- | 9 | 157.23 | 2.22 |
| P56 | MSU180 | SERT+/+ | 7 | 169.00 | 2.10 |
| P56 | MSU180 | SERT+/- | 9 | 176.12 | 4.45 |
| P56 | MSU180 | SERT-/- | 9 | 157.81 | 4.77 |
| P56 | MSUS180 | SERT+/+ | 8 | 164.51 | 3.19 |
| P56 | MSUS180 | SERT+/- | 9 | 171.52 | 6.70 |
| P56 | MSUS180 | SERT-/- | 8 | 163.16 | 4.97 |
| P63 | CTR | SERT+/+ | 10 | 183.48 | 4.24 |
| P63 | CTR | SERT+/- | 9 | 184.96 | 3.62 |
| P63 | CTR | SERT-/- | 7 | 174.40 | 4.61 |
| P63 | MS360 | SERT+/+ | 8 | 185.45 | 1.22 |
| P63 | MS360 | SERT+/- | 9 | 189.74 | 3.16 |
| P63 | MS360 | SERT-/- | 8 | 169.51 | 5.01 |
| P63 | MS180 | SERT+/+ | 9 | 196.18 | 3.96 |
| P63 | MS180 | SERT+/- | 9 | 180.67 | 5.21 |
| P63 | MS180 | SERT-/- | 9 | 174.09 | 1.95 |
| P63 | MSU180 | SERT+/+ | 7 | 180.97 | 1.92 |
| P63 | MSU180 | SERT+/- | 9 | 191.12 | 4.67 |
| P63 | MSU180 | SERT-/- | 7 | 170.47 | 1.35 |
| P63 | MSUS180 | SERT+/+ | 8 | 180.19 | 2.87 |
| P63 | MSUS180 | SERT+/- | 9 | 186.38 | 7.10 |
| P63 | MSUS180 | SERT-/- | 8 | 178.07 | 5.86 |
| P70 | CTR | SERT+/+ | 9 | 186.96 | 2.42 |
| P70 | CTR | SERT+/- | 9 | 198.01 | 4.33 |
| P70 | CTR | SERT-/- | 7 | 184.57 | 4.93 |
| P70 | MS360 | SERT+/+ | 9 | 196.24 | 2.71 |
| P70 | MS360 | SERT+/- | 9 | 202.42 | 2.60 |
| P70 | MS360 | SERT-/- | 8 | 180.07 | 5.11 |
| P70 | MS180 | SERT+/+ | 9 | 204.54 | 3.73 |
| P70 | MS180 | SERT+/- | 9 | 192.77 | 5.70 |
| P70 | MS180 | SERT-/- | 9 | 181.24 | 2.56 |
| P70 | MSU180 | SERT+/+ | 7 | 194.76 | 2.47 |
| P70 | MSU180 | SERT+/- | 9 | 199.42 | 4.88 |
| P70 | MSU180 | SERT-/- | 9 | 182.87 | 3.74 |
| P70 | MSUS180 | SERT+/+ | 8 | 192.88 | 3.83 |
| P70 | MSUS180 | SERT+/- | 9 | 196.53 | 6.51 |
| P70 | MSUS180 | SERT-/- | 8 | 187.43 | 5.76 |

**Suppl. Table S3.** Final sample sizes (*n*), means, and SEMs for offspring sucrose preference (%) and sucrose intake (mg/gr rat) across experimental groups.

| **Sucrose**  **concentration** | **MS_type** | **genotype** | **n** | **Sucrose**  **Preference** |  | **Sucrose**  **intake** |  |
| --- | --- | --- | --- | --- | --- | --- | --- |
|  |  |  |  | **mean** | **sem** | **mean** | **sem** |
| 0.25% | CTR | SERT+/+ | 10 | 59.81 | 8.89 | 0.17 | 0.04 |
|  | MS360 | SERT+/+ | 8 | 67.29 | 9.49 | 0.21 | 0.03 |
|  | MS180 | SERT+/+ | 9 | 55.64 | 12.89 | 0.18 | 0.05 |
|  | MSU180 | SERT+/+ | 7 | 81.84 | 9.34 | 0.25 | 0.05 |
|  | MSUS180 | SERT+/+ | 6 | 74.34 | 14.20 | 0.3 | 0.06 |
|  | CTR | SERT+/- | 9 | 74.8 | 9.53 | 0.31 | 0.09 |
|  | MS360 | SERT+/- | 8 | 47.54 | 10.03 | 0.15 | 0.03 |
|  | MS180 | SERT+/- | 9 | 68.88 | 10.86 | 0.23 | 0.04 |
|  | MSU180 | SERT+/- | 8 | 74.21 | 9.72 | 0.27 | 0.03 |
|  | MSUS180 | SERT+/- | 9 | 56.84 | 9.44 | 0.17 | 0.03 |
|  | CTR | SERT-/- | 6 | 64.37 | 14.84 | 0.23 | 0.05 |
|  | MS360 | SERT-/- | 7 | 70.34 | 6.49 | 0.31 | 0.05 |
|  | MS180 | SERT-/- | 9 | 77.65 | 7.48 | 0.29 | 0.03 |
|  | MSU180 | SERT-/- | 9 | 71.93 | 12.50 | 0.23 | 0.04 |
|  | MSUS180 | SERT-/- | 9 | 66.04 | 11.24 | 0.21 | 0.04 |
| 0.50% | CTR | SERT+/+ | 9 | 74.92 | 10.37 | 0.46 | 0.08 |
|  | MS360 | SERT+/+ | 9 | 74.6 | 5.49 | 0.55 | 0.07 |
|  | MS180 | SERT+/+ | 8 | 79.41 | 9.11 | 0.5 | 0.06 |
|  | MSU180 | SERT+/+ | 7 | 70.14 | 9.60 | 0.45 | 0.07 |
|  | MSUS180 | SERT+/+ | 7 | 89.3 | 4.73 | 0.67 | 0.08 |
|  | CTR | SERT+/- | 9 | 78.61 | 9.79 | 0.5 | 0.09 |
|  | MS360 | SERT+/- | 9 | 82.34 | 7.16 | 0.52 | 0.10 |
|  | MS180 | SERT+/- | 8 | 88.35 | 3.91 | 0.58 | 0.09 |
|  | MSU180 | SERT+/- | 9 | 79.19 | 7.51 | 0.56 | 0.08 |
|  | MSUS180 | SERT+/- | 8 | 84.88 | 6.14 | 0.7 | 0.10 |
|  | CTR | SERT-/- | 7 | 60.9 | 10.72 | 0.48 | 0.08 |
|  | MS360 | SERT-/- | 8 | 75.8 | 6.65 | 0.71 | 0.09 |
|  | MS180 | SERT-/- | 9 | 90.29 | 2.50 | 0.73 | 0.04 |
|  | MSU180 | SERT-/- | 8 | 72.35 | 10.29 | 0.7 | 0.21 |
|  | MSUS180 | SERT-/- | 9 | 70.26 | 9.65 | 0.46 | 0.09 |
| 0.75% | CTR | SERT+/+ | 8 | 80.56 | 8.23 | 0.83 | 0.16 |
|  | MS360 | SERT+/+ | 9 | 84.39 | 7.41 | 1.15 | 0.25 |
|  | MS180 | SERT+/+ | 9 | 75.46 | 6.98 | 0.91 | 0.17 |
|  | MSU180 | SERT+/+ | 7 | 90.32 | 4.61 | 1.03 | 0.22 |
|  | MSUS180 | SERT+/+ | 6 | 65.64 | 14.85 | 0.95 | 0.30 |
|  | CTR | SERT+/- | 9 | 65.42 | 10.99 | 0.8 | 0.21 |
|  | MS360 | SERT+/- | 9 | 74.66 | 9.14 | 0.8 | 0.17 |
|  | MS180 | SERT+/- | 8 | 86.34 | 10.21 | 1.1 | 0.23 |
|  | MSU180 | SERT+/- | 9 | 72.56 | 11.72 | 0.89 | 0.20 |
|  | MSUS180 | SERT+/- | 9 | 91.42 | 2.00 | 1.37 | 0.11 |
|  | CTR | SERT-/- | 6 | 76.19 | 14.05 | 1.22 | 0.31 |
|  | MS360 | SERT-/- | 8 | 81.7 | 8.39 | 1.44 | 0.30 |
|  | MS180 | SERT-/- | 9 | 81.07 | 7.16 | 1.15 | 0.18 |
|  | MSU180 | SERT-/- | 9 | 89.11 | 3.12 | 1.38 | 0.29 |
|  | MSUS180 | SERT-/- | 9 | 75.37 | 8.51 | 1.08 | 0.21 |
| 1.00% | CTR | SERT+/+ | 9 | 94.75 | 1.51 | 1.73 | 0.32 |
|  | MS360 | SERT+/+ | 9 | 86.14 | 6.54 | 1.9 | 0.23 |
|  | MS180 | SERT+/+ | 9 | 85.94 | 6.40 | 1.44 | 0.17 |
|  | MSU180 | SERT+/+ | 7 | 89.01 | 4.66 | 1.44 | 0.24 |
|  | MSUS180 | SERT+/+ | 7 | 92.8 | 5.84 | 2.34 | 0.27 |
|  | CTR | SERT+/- | 8 | 89.19 | 5.66 | 1.47 | 0.18 |
|  | MS360 | SERT+/- | 8 | 93.3 | 3.00 | 2.04 | 0.26 |
|  | MS180 | SERT+/- | 9 | 91.95 | 2.91 | 2.02 | 0.58 |
|  | MSU180 | SERT+/- | 9 | 88.67 | 3.49 | 1.52 | 0.36 |
|  | MSUS180 | SERT+/- | 8 | 95.63 | 0.88 | 1.94 | 0.18 |
|  | CTR | SERT-/- | 5 | 95.88 | 1.99 | 2.14 | 0.31 |
|  | MS360 | SERT-/- | 7 | 76.65 | 7.76 | 2.12 | 0.47 |
|  | MS180 | SERT-/- | 9 | 86 | 9.73 | 1.99 | 0.27 |
|  | MSU180 | SERT-/- | 7 | 85.19 | 10.50 | 1.62 | 0.26 |
|  | MSUS180 | SERT-/- | 9 | 96.3 | 1.18 | 1.96 | 0.10 |

*Note: Reported sample sizes reflect final animal counts per group after excluding animals lost due to leaking bottles or incorrect data entry of bottles’ weight*
